# Walnut intake, cognitive outcomes and risk factors: a systematic review and meta-analysis

**DOI:** 10.1080/07853890.2021.1925955

**Published:** 2021-06-16

**Authors:** Danielle Cahoon, Shruti P. Shertukde, Esther E. Avendano, Jirayu Tanprasertsuk, Tammy M. Scott, Elizabeth J. Johnson, Mei Chung, Nanguneri Nirmala

**Affiliations:** a Gerald J. and Dorothy R. Friedman School of Nutrition Science and Policy, Tufts University, Boston, MA, USA; b Department of Public Health and Community Medicine, Tufts University School of Medicine, Boston, MA, USA; c Center for Clinical Evidence Synthesis, Institute for Clinical Research and Health Policy Studies, Tufts Medical Center, Boston, MA , USA

**Keywords:** Walnut, cognition, cognitive decline, mood, depression, stroke, inflammation, HbA1c, HOMA-IR, glucose metabolism

## Abstract

**Background:**

Walnuts contain nutrients that are associated with improved cognitive health. To our knowledge, no review has systematically examined the effects of walnuts on cognitive function and risk for cognitive decline.

**Objective:**

To conduct a systematic review and meta-analysis evaluating the effects of walnut intake on cognition-related outcomes and risk-factors for cognitive decline in adults.

**Methods:**

Medline^®^, Commonwealth Agricultural Bureau, and Cochrane Central Register of Controlled Trials were searched for randomized controlled trials (RCTs) and observational studies published until April 2020 on walnut intake, cognition (e.g. cognitive function, stroke, and mood), and selected risk factors for cognitive decline (e.g. glucose homeostasis and inflammation). Risk-of-bias and strength-of-evidence assessments were conducted using standard validated tools. Random-effects meta-analyses were conducted when ≥3 studies reported quantitative data for each outcome.

**Results:**

32 RCT and 7 observational study publications were included. Meta-analysis of cognition-related outcomes could not be conducted due to heterogeneity of tests. None of the 5 cognition RCTs found significant effects of walnuts on overall cognition, although 3 studies found improvements on subdomains and/or subgroups. All 7 observational studies found significant associations and a dose-response relationship between walnut intake and cognition-related outcomes. Meta-analyses of 27 RCTs reporting glucose homeostasis and inflammation outcomes, selected risk factors for cognitive decline, did not show significant effects of walnut intake.

**Conclusions:**

Due to the non-uniformity of tests for cognition-related outcomes, definitive conclusions regarding the effect of walnut consumption on cognition could not be reached. Additionally, evidence does not show associations between walnut intake and glucose homeostasis or inflammation, cognitive decline risk-factors. High-quality studies with standardized measures are needed to clarify the role of walnuts in cognitive health.

**KEY MESSAGES**
- This is a systematic review and meta-analysis of 5 randomized clinical trials and 7 observational study articles of the impact of walnut intake on cognition decline and 27 randomized clinical trials of the effect of walnut intake on risk factors for cognitive decline including glucose homeostasis and inflammation.
- The non-uniformity of tests performed to measure cognitive function in the various studies did not allow for a meta-analysis of these studies. A definitive conclusion could therefore not be reached regarding the effect of walnut intake on cognitive decline.
- The evidence available does not show an association between walnut intake and glucose homeostasis or inflammation.

## Introduction

The prevalence of age-related cognitive decline is expected to grow with increases in life expectancy [1,2]. Additionally, risk factors for cognitive decline, including vascular and metabolic disorders [3,4] continue to rise at unparalleled rates [1]. The projected increases in personal and public health burdens raise a need to identify strategies to promote cognitive health and reduce risk factors for cognitive decline [4]. Nutritional interventions could be adopted as inexpensive and accessible strategies to reduce the risk of cognitive decline.

Walnuts contain nutrients that may intervene in the development of cognitive decline, in part by targeting cardiometabolic risk factors [5]. These nutrients include essential fatty acids, soluble fiber, vitamin E, and polyphenols (e.g. ellagitannins) [5], which individually or in combination may produce beneficial effects on serum lipids, [6] blood pressure, oxidative stress, and inflammation [7–10]. Given the role of vascular and metabolic disturbances in cognitive decline [3,4], the aforementioned biological mechanisms suggest that incorporating walnuts into regular diet can promote cognitive health. Additionally, intakes of nutrients in walnuts such as omega-3 fatty acid α-linolenic acid, dietary fiber, vitamin E, vitamin B6, folate, potassium, and polyphenols have been associated with improved cognitive function [11–16]. Furthermore, animal studies have shown that walnut supplementation attenuates age-related declines in cognitive function [2].

While a role for walnuts in promoting cognitive health has been previously reviewed [2,17], to our knowledge, a systematic evaluation of this role in humans has not been conducted. Therefore, in this publication, we summarized the existing evidence of the impact of walnut intake on cognition with a systematic review of randomized controlled trials (RCTs) and observational studies. We also review and meta-analyze the evidence onthe effects of walnut consumption on selected risk factors for cognitive decline.

## Methods

This systematic review was conducted following standard methodology outlined in the Cochrane Handbook [18] and the results are reported according to the Preferred Reporting Items for Systematic Reviews and Meta-Analyses (PRISMA) statement [19]. During the scoping phase of this systematic review, a technical expert panel was convened that served as key informants to refine key questions,walnut interventions, and cognition-related outcomes of interest.

### Data sources and literature search strategy

We conducted electronic searches on Medline^®^, Commonwealth Agricultural Bureau (CAB), and Cochrane Central Register of Controlled Trials from 1946 to April 2020. The search strategy included terms for walnut interventions of interest and outcomes of interest (described later) and was limited to human studies and the English language. The complete search strategy is shown in Supplemental Table S1. The reference lists of prior relevant systematic reviews were screened to identify additional eligible studies not captured by the database searches.

### Study eligibility criteria

#### Inclusion criteria

We included all interventional and observational studies in adults (≥18 years) who were healthy or at increased risk for cognitive decline. Walnut interventions of interest included whole walnuts, walnut oil, or walnut extract. The primary outcomes of interest were cognition-related outcomes (e.g. cognitive function, dementia, cerebrovascular diseases, brain imaging, mood, anxiety, depression). Secondary outcomes included risk factors for cognitive decline. With input from key informants, we identified major risk factors for cognitive-decline as alterations in blood lipids, glucose metabolism, blood pressure, endothelial function, inflammation, and oxidative stress [2–4,10]. Since systematic reviews and meta-analyses examining the effects of walnuts on blood lipids, blood pressure, oxidative stress [20] as well as endothelial function [21] have been published recently, we included glucose homeostasis and inflammation outcomes in this review. In addition, for interventional studies, we set the minimum intervention duration for inclusion according to specific outcomes of interest: any duration for inflammation and mood outcomes, ≥ 1 week for glucose outcomes, and ≥ 3 weeks for all other outcomes. For publications reporting on the same trial and outcome of interest, but at different time points, only the publication with the longer intervention duration was retained.

#### Exclusion criteria

We excluded studies that compared interventions in which the effects of walnuts could not be isolated (e.g. walnuts mixed with other nuts), or did not correlate walnut consumptions with any one of the outcomes of interest. Detailed study eligibility criteria are listed in Table 1.

**Table 1.** Study Eligibility Criteria^a^.

Population	Adult*s* ≥ 18 years
	Healthy
	At risk for increasing cognitive decline (e.g. those with baseline obesity, hyperlipidaemia, hypertension, diabetes, or metabolic syndrome)
Intervention	Walnut
	Walnut oil
	Walnut extract
Comparator	No walnut
	Lower dose of walnut
	Other nuts/foods
Outcome	Cognitive function
	Dementia
	Alzheimer’s disease and pathology
	Mild cognitive impairment
	Cerebrovascular disease (e.g. stroke)
	Brain imaging
	Mood (e.g. anxiety, depression)
	Glucose metabolism (e.g. HbA1c and HOMA-IR)
	Inflammation (e.g. hsCRP, IL-1β, IL-6 TNFɑ, E-selectin, sICAM-1, sVCAM-1)
Design	All designs except case reports
Duration of exposure (RCTs only)	Any duration: inflammatory markers and mood
	≥1 week intervention: glucose outcomes
	≥3 week intervention: all other outcomes

^a^HbA1C: haemoglobin A1C; HOMA-IR: homeostatic model assessment-insulin resistance; hsCRP: high sensitivity c-reactive protein; IL: interleukin; RCT: randomized-controlled trial; sICAM-1: soluble intracellular adhesion molecule-1; sVCAM-1: soluble vascular cell adhesion molecule-1; TNFɑ: tumour necrosis factor-alpha; USA: United States of America.

### Study selection process

Titles and abstracts identified from the literature searches were independently screened by two investigators to exclude irrelevant citations using the open-source online software Abstrackr (http://abstrackr.cebm.brown.edu/). For all abstracts deemed potentially relevant by both screeners, full-text articles were retrieved and independently screened by two investigators based on the study eligibility criteria. All abstract and full-text screening conflicts or disagreements were resolved through discussions between the two investigators or through the consensus of the entire research team. Excluded articles and reasons for exclusion are listed in Supplemental Table S3.

### Data extraction (stages 1 and 2)

We used a two-stage approach for data extractions. During the scoping stage (stage 1) of the systematic review, we extracted information from all included studies about study design, types of exposure/interventions, and outcomes reported in each included study. Due to the large number of inflammation and glucose homeostasis measures investigated across studies, we set aside (i.e. not performing the next stage of data extraction) the measures if they were reported in 2 or fewer studies.

For the second-stage data extraction, we designed customized Excel data extraction forms for interventional and observational studies. The items extracted included the following: funding sources, location, study population characteristics, enrolled and analysed sample sizes, study design features, walnut intake doses (all doses converted to grams for walnuts and mL for walnut oil), relevant outcomes assessed and methods used, confounders and effect modifiers used in statistical analysis, and results. For interventional studies, we additionally extracted information on walnut interventions and comparators, adherence, and adverse effects. For studies with multiple walnut-free comparator arms, the intervention that was most similar to the walnut group was chosen as the comparator for our analyses. Quantitative results that were needed for meta-analysis were extracted wherever provided, otherwise, qualitative results were extracted (i.e. direction of association and statistical significance). For all studies, results from the most adjusted statistical model were extracted in preference over crude or age-adjusted measures. The extraction forms were piloted on several studies and revised according to group discussions. Data from each study were extracted by one investigator and confirmed by ≥1 other investigator. Data discrepancies were identified and resolved through group discussions.

### Risk of bias in individual studies

For each included study, two independent investigators conducted risk of bias (ROB) assessments and discrepancies were resolved *via* group discussion. For interventional studies, we used the Cochrane risk of bias (ROB 2.0) tool [22] to evaluate risk of bias in the following five domains: randomization process (e.g. sequence generation, allocation concealment), deviations from intended interventions (e.g. blinding of participants and researchers, occurrence and balance of deviations between groups, appropriateness of analysis), missing outcome data, measurement of outcome (e.g. method used, masking of outcome assessment), and selective outcome reporting. As specified by the tool, each domain was individually graded for risk of bias as low, some concerns, or high. Overall risk of bias was determined through consensus between the two investigators.

The ROB of prospective cohort studies was assessed using the Newcastle Ottawa Scale (NOS) for cohort studies [23]. The NOS uses a scoring system of stars to evaluate risk of bias in three domains: selection (e.g. representativeness of cohort, selection of non-exposed cohort, ascertainment of exposure), comparability (e.g. control for important factors on basis of design or analysis), and outcome (e.g. appropriate method and follow-up). A modified version of the NOS was used to assess ROB for cross-sectional studies [24,25] **(**Supplemental File 1). The maximum number of stars was 9 for prospective cohort studies and 10 for cross-sectional studies. Following the criteria used in previous reviews, 0–4 stars was considered high risk of bias, 5–7 was some concerns, and 8–9 (prospective cohort) or 8–10 (cross-sectional) was considered low risk of bias [24,25].

### Data synthesis and meta-analysis

All of the included studies were summarized in narrative form and in the summary tables that tabulated key features of the study populations, design, intervention, outcomes, and results. Summary tables were organized by study type (i.e. RCTs and observational studies) and by outcomes of interest (cognition-related outcomes and outcomes related to risk-factors for cognitive decline).

We did not perform meta-analyses to quantitatively synthesize the results from observational studies because these studies used a wide variety of outcome measures and no outcome measure of interest was reported in more than two studies. For RCTs, we performed random-effects meta-analyses where there were ≥ 3 unique studies reporting the same outcomes in light of clinical heterogeneity (different doses and types of walnut interventions). We used the reported or calculated net change (difference of the 2 within-group changes from baseline) between the walnut and comparison groups as the effect size measure in the meta-analysis. If the standard deviations (SDs) of the within-group changes were not reported, the SD of the mean within-group change was estimated by using the following formula: SD_diff_ = √( SD_Base_+ SD_final_ − 2 x Corr x SD_Base_ x SD_Final_), where SD_Base_ is the SD at baseline and SD_final_ is the SD at the end of the intervention. We assumed a correlation coefficient (Corr) value of 0.50 to impute the missing SD of the mean within-group change. Sensitivity analyses that used Corr values of 0.20 and 0.80 were conducted to evaluate the impacts of the correlation assumptions on the meta-analysis results, and none showed appreciable impacts on the pooled results. Studies were excluded from meta-analyses if the required information for the aforementioned calculations was not reported, not standardisable, or otherwise implausible for any given outcome. We used both the Q statistic (considered significant when *p* < .10) and the I^2^ index to quantify the extent of statistical heterogeneity [18]. Low, moderate, and high heterogeneity was defined as I^2^ values below 25%, between 25 and 70%, and above 70%, respectively.

All calculations and meta-analyses were conducted using Stata SE 13 (StataCorp). Two-tailed *p* values ≤ .05 were considered significant. The analytic data sets can be found under Supplemental File 2. The authors confirm that the data supporting the findings of this study are available within the article and its Supplementary materials.

### Strength of evidence rating

We followed the Grades of Recommendation, Assessment, Development, and Evaluation (GRADE) approach to determine strength of evidence (SoE) for each outcome [26–28]. Briefly, SoE, consists of the following domains: limitations (i.e. individual study’s ROB), directness of evidence, indirectness, and the precision of effect estimates. We added “dose-response” domain as an upgrading factor if a dose-response relationship between walnut intake and cognition or risk factor outcomes exists. Based on the assessment across these domains, an overall SoE rating of very low, low, moderate, or high was assigned for each outcome. This process was executed by consensus among all investigators.

## Results

**Figure 1.**
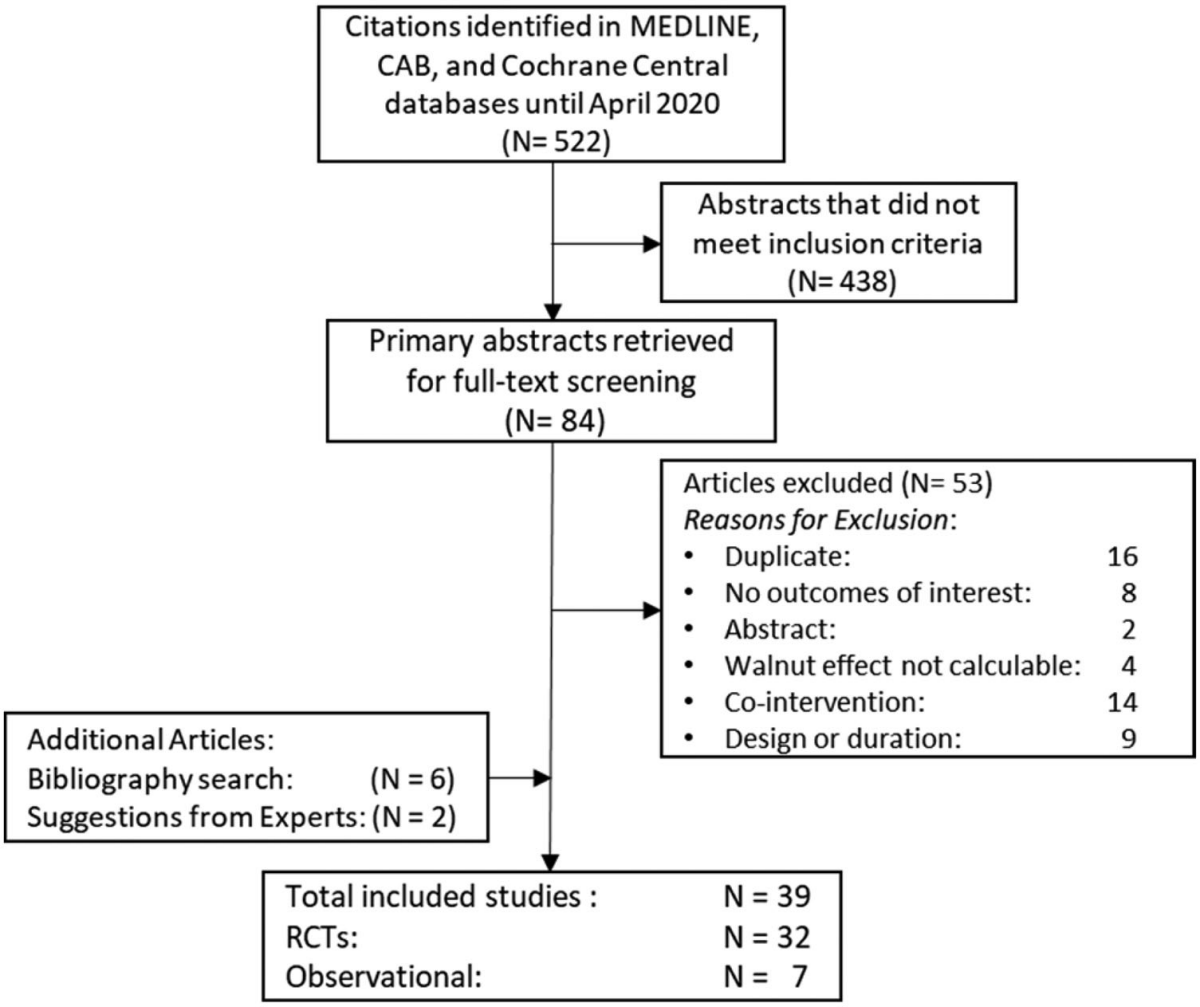
Study flow diagram showing the number of abstracts identified (*n* = 522); abstracts not meeting criteria (*n* = 438); full-text articles retrieved (*n* = 84); full-text articles excluded after screening (*n* = 53); full-text articles added from grey literature search (*n* = 6); full-text articles suggested by key informants (*n* = 2); full-text articles meeting study eligibility criteria (*n* = 39); eligible randomized controlled trials (*n* = 32), and observational studies (*n* = 7).

The results of the search strategy for overall inclusion in this systematic review and meta-analysis can be found in Figure 1, which includes 5 RCTs and 7 observational studies with cognition-related outcomes, and 27 RCTs with outcomes related to glucose metabolism and/or inflammation.

### Cognition-related outcomes

Seven observational studies (6 cross-sectional studies [20,29–33], 1 prospective cohort study [34]) and five RCTs [35–39] examining the associations between walnut consumption and cognition-related outcomes (cognitive function, brain MRI, stroke, mood) were identified. Cognition-related outcome domains, subdomains, and measurement tools were highly variable across the included studies. As a result, it was not possible to perform meta-analyses for cognition-related outcomes. Results of RCTs and observational studies reporting the cognition-related outcomes are summarized below in three main categories – cognitive function, mood and stroke.

### Cognitive function

#### RCTs

Two randomized (1 cross-over [35] and 1 parallel [37]) trials reporting the effects of walnut on cognitive function were included. One study was conducted in generally healthy young adults, while the other study examined older subjects with mixed baseline health (healthy or with type 2 diabetes [T2D], hypertension [HTN], and/or hyperlipidaemia [HLD]) (Table 2). Despite differences in walnut interventions, study populations, and cognitive tests, neither study found a significant overall effect of walnut on composite scores for global cognition and/or cognitive domains. However, both studies reported significant effects of walnuts on subdomains of cognition and/or subgroups of the study population.

**Table 2.** Study characteristics and key findings of RCTs reporting the effects of walnut on cognition-related outcomes^a^.

Study (Year)	Country (funding); design	Walnut intervention	Control intervention^b^	Baseline health (%)^c^	Mean BMI (SD)	*N* Randomized (*N* analyzed)	Mean age (SD), years	% Male	Washout period	Total duration (exposure duration)	Cognition-related outcomes (measurement tool(s))	Key findings	ROB^e^
Miller et al. (2018) [38]	England (Gov, Aca); RP	30 mL walnut oil in milkshake	Milkshake with no walnut	Healthy (100), Overweight (17)	22.5 (NR)	62 (59)	19.9 (2.5)	37.2	NA	100 mins (NA)^d^	Mood (POMS)	No significant difference between walnut and control on POMS.	High
Pribis et al. (2012) [35]; (2016) [36]^f^	USA (Ind); RCO	60 g/d walnuts in banana bread	Banana bread with no walnuts	Healthy (100), Overweight/ Obese (26.6)	22.9 (3.4)	64 (52)	20.7 (2.1)	NR	6 wks	22 wks (8 wks)	Non-verbal Reasoning (APM) Memory (WMS-III) Verbal Reasoning (WGCTA) Mood (POMS [TMD])	No significant differences on APM or WMS-III. WGCTA: walnut improved performance only on interference subtest vs. control (11.2%, *p* = .009, *d* = 0.567). Walnut improved TMD score in males only (−27.49%, *p* = .043, *d* = 0.708).	SC
Probst et al. (2012) [39]	Austria (NR); RCO	41.6 g/d walnuts in 300 g of 1% fat yogurt	300 g of 1% fat yogurt	Healthy (100), Obese (0)	23.1 (NR)	14 (14)	24 (NR)	100	1 wk	6 wks (15 mins)^d^	Mood (MDMQ [fatigue, calmness, mood])	No significant difference between walnut and control on MDMQ.	High
Sala-Vila *et al.* (2020) [37]	USA, Spain (Gov, Ind, Aca); RP	30–60 g/d walnuts	No walnuts	T2D (9.6), HTN (52.2), HLD (54), Overweight/ Obese (NR)	27.3 (NR)	708 (657)	69.2 (NR)	33	NA	2 yrs (2 yrs)	*Composite Scores for:*Memory (RAVLT, ROCF) Language (semantic fluency, BNT) Perception (VOSP, WAIS-III) Frontal Function (TMT [A, B], FAS, SCWT, SDMT, Digit span [WAIS-III], CPT-III) Global Cognition (all tests above) Structural Brain MRI (T1-weighted) fMRI (during N-back test)	No significant differences between walnut and control on any composite score (adjusted mean change). In Barcelona subgroup, walnut improved global cognition (*p* = .016) and perception (*p* = .005) scores. No significant differences in Loma Linda subgroup. Structural MRI: no significant difference between walnut and control. fMRI: at 2 yrs vs. baseline, increased activation in control group, but not walnut group (suggests ↑ brain efficiency in walnut group).	High

^a^Aca: Academia; APM: Raven’s Advanced Progressive Matrices; BMI: Body Mass Index (kg/m^2^); BNT: Boston Naming Test; CPT-II: Conners Continuous Performance Test-II; FAS: Phonemic fluency test; fMRI: Functional MRI; Gov: Government; HLD, hyperlipidaemia (includes hypercholesterolemia); HTN: hypertension; Ind: industry; MDMQ; Multidimensional Mood State Questionnaire; MRI: Magnetic Resonance Imaging; *N*: number; NA: not applicable; NR: not reported; POMS: Profile of Mood States Questionnaire; RAVLT: Rey Auditory Verbal Learning Test; RCO: randomized cross-over; RCT: randomized-controlled trial; ROB: risk of bias; ROCF: Rey-Osterrieth Complex Figure; RP: randomized parallel; SC: some concerns; SCWT: Stroop Colour Word Test; SD: standard deviation; SDMT: Symbol Digit Modalities Test; T2D: type-II diabetes; TMD: Total Mood Disturbance Score; TMT: Trail Making Test; Part A and B; USA: United States of America; VOSP: Visual Object and Space Perception Battery; WAIS-III: Wechsler Adult Intelligence Scale; WGCTA: The Watson-Glaser Critical Thinking Appraisal; WMS-III: Wechsler Memory Scale III.

^b^For studies with multiple walnut-free intervention arms, the intervention most similar to the walnut intervention was chosen as the control group.

^c‘^Healthy’: no chronic illness (e.g. T2D, MetS, cardiovascular disease, etc.); ‘Health condition (NR)’: unspecified proportion of population has condition.

^d^Intervention was a single dose.

^e^ROB: Overall risk of bias assessment.

^f^Same trial, but reported different outcomes.

**Figure 2.**
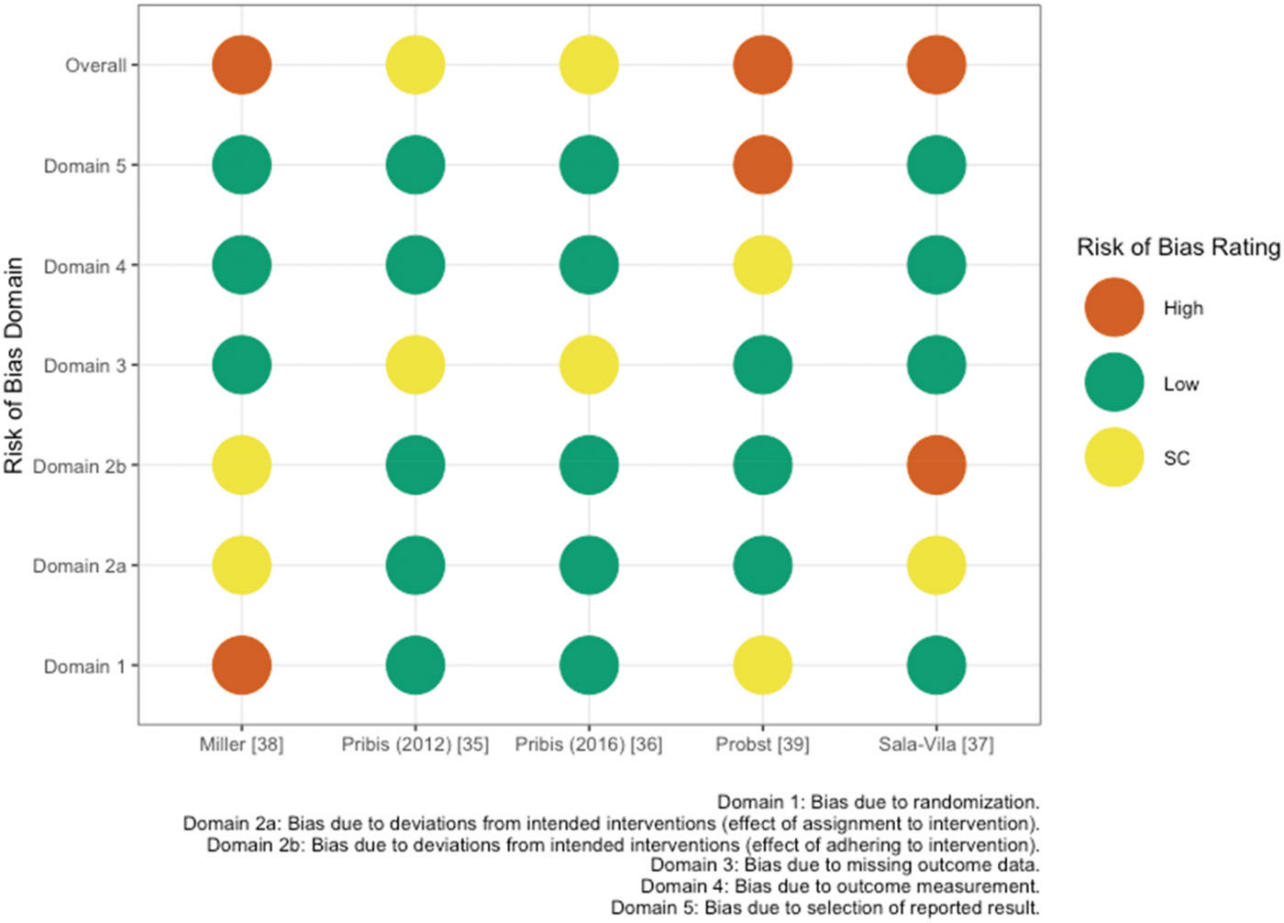
Risk of bias assessment based on the *Cochrane risk-of-bias tool for randomized trials (RoB 2)* conducted for five publications reporting cognition and mood outcomes. SC: some concerns.

In healthy young adults consuming 60 g/d walnuts or placebo for 8 weeks in a cross-over design, Pribis et al. [35] found no significant differences in total scores for memory (WMS-III), non-verbal reasoning (APM), or verbal reasoning (WGCTA). However, walnut consumption significantly improved performance on the inference subtest of the WGCTA relative to placebo (11.2% difference, *p* = .009). Risk of bias for this study was rated as some concerns, due to missing data (Figure 2; Additional information can be found in Supplemental Table S2).

In the Walnut and Healthy Aging Study (WAHA), comprised of older adults (mean age 69.2) with mixed baseline health, Sala-Vila et al. examined the effects of consuming 30–60 g/d walnuts on cognitive function using composite scores for memory (RAVLT, ROCF), language (semantic fluency test, BNT), perception (VOSP, WAIS-III), frontal function (TMT, FAS, SCWT, SDMT, digit span [WAIS-III], CPT-III), and global cognition (all tests) [37] (Table 2). No significant differences were observed between walnut and control groups in adjusted mean change from baseline for all composite scores (Table 3). However, post hoc analyses showed that among subjects from the Barcelona site, there were significant between-group differences in adjusted mean change for global cognition (*p* = .016) and perception (*p* = .005) composite scores, in that the walnut group had less decline in performance than did the control group over the two-year intervention period. No significant differences were observed between groups in subjects from the Loma Linda site. The authors noted that Barcelona participants had significantly lower education and baseline dietary and red blood cell ALA and ALA intake than their Loma Linda counterparts.

**Table 3.** Effects of walnut on cognition-related outcomes reported in five RCTs^a^.

Study (Year)	Group comparisons	Cognition-related outcome	Estimate (95% CI)^b^
Sala-Vila et al. (2020) [37]	Walnut vs. Control	Global Cognition	0.02 (–0.02, 0.06)
		Memory	0.00 (–0.08, 0.09)
		Language	−0.01 (–0.09, 0.07)
		Perception	0.04 (–0.06, 0.14)
		Frontal Function	0.01 (–0.04, 0.06)
Pribis et al. (2012) [35]	Walnut vs. Placebo	General intellectual capacity/non-verbal reasoning	0.20 (–0.80, 1.00)
		Verbal reasoning	1.60 (–2.20, 5.50)
		General memory	−1.30 (–3.90, 1.20)
		Working memory	−0.20 (–1.20, 0.80)
Pribis et al. (2016) [36]	Walnut vs. Placebo	Total Mood Disturbance (Combined Sexes)	−4.40 (–12.3, 3.50)
		Total Mood Disturbance (Males)	−11.0 (–33.13, 11.13)
		Total Mood Disturbance (Females)	0.10 (–15.42, 15.62)
Miller et al. (2018) [38]	Walnut vs. Control	Total Mood Disturbance	−4.99 (–10.75, 0.07)
Probst et al. (2018)[39]	Walnut vs. Control	Mean AUC Values of Mood	0.40 (NR)

^a^AUC: area under the curve; CI: confidence interval; NR: not reported; RCT: randomized-controlled trial.

^b^Mean differences are reported, with the corresponding 95% CIs.

In addition to neuropsychological testing, Sala-Vila et al. conducted structural and functional MRIs to examine brain structure, resting state connectivity, blood flow, and the expression of functional brain networks during cognitive demands (N-back test) in the Barcelona subset [37]. No significant differences were observed between walnut and control groups on structural outcomes. However, findings from fMRIs revealed significantly increased activation during the N-back test in the control group at two years compared to baseline. No changes from baseline were observed in the walnut group, suggesting improved brain efficiency after two years of walnut consumption. Risk of bias for this study was rated as high, due to unblinding of participants and dietitians and deviations from the intended interventions.

#### Cohort & cross-sectional studies

Four observational studies (3 cross-sectional [30–32], 1 prospective cohort [34]) reporting the association between walnut consumption and cognitive function were included (Table 4). Study populations included older adults (mean age: 56.4–74.3) with mixed baseline health (various proportions of overweight, obese, T2D, HLD and/or HTN subjects). One study examined females only [34], and the other three assessed populations with relatively equal proportions of males and females [30–32]. Findings were consistent, as all four studies found significant associations between walnut intake and various measures of cognitive function.

**Table 4.** Study characteristics and key findings of cross-sectional (*n* = 6) and prospective cohort studies (*n* = 1) reporting cognition-related outcomes^a^.

Study (Year)	Country (Funding); Design	Study Name (Enrollment Years)	N Enrolled (N Analysed)	Baseline Health (%)^b^	Mean BMI (SD)	Mean Age (SD), Years	% Male	Walnut Intake Amount(s)	Adjustments	Cognition-related Outcomes (Measurement Tool(s))	Key Findings	ROB^c^
Arab et al. (2015) [30]	USA (Ind); Cross-sectional	NHANES III (1988–1994); NHANES (1999–2002)	20,050 (12,693)	Overweight (NR)	26.8 (NR)	56.4 (NR)	44.7	*NRNC*: 0 g/d**Tertiles for: *WWHC:* >0 to 5; >5 to 11; >11 g/d *WWON:* > 0 to 4; > 4 to 7.7; > 7.7 g/d	Age, sex, race, PA, SMK, ALC	NHANES III Age 20–59: *All subtests of NES2:*Visuomotor Speed (SRTT), Information Processing Speed Concentration, Motor Control (SDST), Learning and Recall (SDLT); NHANES III Age 60+: Attention and Verbal Memory (SRT); NHANES 60+: Attention, Visual Spatial Skills, Learning, Memory (DSST)	Higher walnut intake associated with better scores on: SRTT: *p*-trend <.001 SDST: *p*-trend = .003 SDLT: *p*-trend = .004 SRT: *p*-trend <.001 DSST: *p*-trend <.001	SC
Arab et al. (2019) [29]	USA (Ind); Cross-sectional	NHANES (1988–1994)	50,965 (26,656)	Overweight (NR)	26.8 (NR)	46 (NR)	49	*NN, ON*: 0 g/d *WWON:* 4 g/d *WWHC*: 24 g/d	Age, sex, race, BMI, SMK, ALC, annual household income, marital status	Depression (PHQ-9)	WWHC and WWON significantly associated with reduced depression scores vs. NN. For WWHC, least squared mean for total scores was 26% lower than NN (*p* < .0001) and 17% lower than ON (*p* = .01). Stronger association among women (32%, *p* < .0001) than men (21%, *p* = .05).	SC
Bishop et al. (2020) [31]	USA (Ind); Cross-sectional	HCNA and HRS (2012–2016)	3,632 (2,821)	Overweight (37.8), Obese (30.2)	NR	74.3 (NR)	42.9	*No intake:* 0 oz/d*Low intake:* 0.04 oz/d, *Moderate intake*: 0.49 oz/d	Age, sex, race, marital and retirement status, EDU, occupation, household income, BMI, PA, SMK, ALC, chronic conditions	Global Cognitive Function (TICS Total Score)	Walnut intake (low or moderate vs. none) was associated with better test scores in 2012, 2014, and 2016 (*p* < .001), but no association was seen between walnut consumption and cognitive change overtime.	Low
Guasch-Ferré et al. (2017) [20]	USA (Gov, Ind); Cross-sectional	NHS (1986–2012); NHS II (1991–2013); HPFS (1986–2012)	289,900 (210,863)	All cohorts: HLD (NR), HTN (NR), T2D (NR)	NR	NR	19.6	*28 g serving;* 0 vs. ≥ 1 servings/wk	Age, race, BMI, PA, SMK, vitamin and aspirin use, family history of T2D, cancer, MI, HTN, HLD, menopausal status, hormone and oral contraceptive use, diet (total energy, ALC, red or processed meat, fruit, vegetable intake)	Fatal and Non-Fatal Stroke (Medical Records)	Walnut consumption was associated with a 17% reduction in risk for stroke (HR: 0.83; 95% CI, 0.71–0.96)	SC
Liu et al. (2020) [33]	USA (Ind); Cross-sectional	NHS (1986–2012); NHS II (1991–2013); HPFS (1986–2012)	289,660 (192,655)	NHS: HTN (26.4), HLD (22.4); NHS II: HTN (7.1), HLD (15.8); HPFS: HTN (17.4), HLD (21.1)	25.4 (NR); 24.7 (NR); 25.4 (NR)	58.4 (NR); 41.3 (NR); 58.2 (NR)	17.7	*28 g serving; *per 0.5 serving/d	Age, race, sex, family history of CVD, calendar year in 4-year intervals, intake of nuts at each 4-year interval, SMK, ALC, menopausal status, hormone use, oral contraceptive use, PA, BMI, changes in energy intake, HLD, HTN	Fatal and Non-Fatal Stroke (Medical Records)	Per 0.5 servings/d, increased walnut intake during a 4-year interval was associated with a 20% lower risk for stroke (RR: 0.80, 95% CI, 0.67–0.95) in the subsequent 4-year interval.	SC
O’Brien et al. (2014) [34]	USA (Gov, Ind); Prospective cohort	NHS (1995–2001)	19,415 (15,467)	HTN (55.1), HLD (65.3), MI (5.9), T2D (10), Overweight (35), Obese (17.2)	NR	74.2 (NR)	0	*28 g serving* < 1/mo, 1–3/mo, ≥ 1/wk	Age, EDU, time between tests, BMI, anti-depressants, SMK, PA, total energy, ALC, vitamin intake, HTN, HLD, MI, T2D	Cognitive Function (TICS), Verbal Memory Score (average of EBMT [immediate, delayed recall], fluency test [animals], TICS [delayed recall of word list], DS-backward), Global Score (average of all 6 tests)	Significantly higher global cognitive and verbal memory scores in 1–3 servings/mo group vs. < 1 serving/mo group (global score: 0.03, 95% CI: 0.01–0.06; verbal memory score: 0.03, 95% CI: 0.00–0.06). No overall trend of increasing walnut intake with improved cognitive performance and no significant differences between intake groups in rates of cognitive decline over the six years of follow-up.	SC
Valls-Pedret et al. (2012) [32]	Spain (Gov); Cross-sectional	PREDIMED (2004–2009)	578 (447)	T2D (55.9), HLD (72.0), HTN (75.2)	28.5 (NR)	66.9 (NR)	47.9	Per 30 g/d walnut	Age, sex, EDU, BMI, SMK, ApoE e4 allele, PA, T2D, HTN, HLD	Global Cognitive Function (MMSE), Immediate and Delayed Episodic Verbal Memory (RAVLT), Episodic Memory of Performance (VPAT [WMS]), Semantic Fluency (AFT), Immediate Memory (DS-forward [WAIS]), Working Memory (DS-back [WAIS]), Executive Function (CTT-I, II)	Higher walnut intake was associated with better scores on digit span backward test of working memory (*p* = 0.039). Higher intake was not associated with better performance on other measures.	SC

^a^Aca: Academia; AFT: Animal Fluency Test; ALC: alcohol; BMI: Body Mass Index (kg/m^2^); CI: confidence interval (95%); CTT-I; II: Colour Trail Test (parts I and II); CVD: cardiovascular disease (includes coronary artery disease, peripheral vascular disease, congestive heart failure, or carotid stenosis); WWHC: walnuts with high certainty; DS-back: Digit Span Backward; DS-forward: Digit Span Forward; DSST: Digit-Symbol Substitution Test; EBMT: East Boston Memory Test (immediate and delayed recall); EDU: education; Gov: Government; HCNS: Health Care and Nutrition Study; HLD: hyperlipidaemia (includes hypercholesterolemia); HPFS: Health Professionals Follow-up Study; HR: hazard ratio; HRS: Health and Retirement Study; HTN: hypertension; Ind: Industry; MI: myocardial infarction; MMSE: Mini-Mental State Examination; N: number; NA: not applicable; NES2: Neurobehavioral Evaluation System 2; NHANES: National Health and Nutrition Examination Survey; NHS: Nurses’ Health Study; NN: no nuts; NR: not reported; ON: other nuts; oz: ounce; PA: physical activity; PHQ-9: Patient Health Questionnaire – 9; Major Depressive Disorder Module; PREDIMED: Prevención con Dieta Mediterránea; RAVLT: Rey Auditory Verbal Learning Test; ROB: risk of bias; RR: risk ratio; SC: some concerns; SD: standard deviation; SDLT: Serial Digit Learning Test; SDST: Symbol Digit Substitution; SMK: smoking; SRT: Story Recall Test; SRTT: Simple Reaction Time; T2D: type-II diabetes; TICS: Telephone Interview of Cognitive Status; USA: United States of America; VPAT: Verbal Paired Associates Test (WMS subtest); WAIS: Wechsler Adult Intelligence Scale; WMS,: Wechsler Memory Scale; WWON: walnuts with other nuts.

^b^Health condition (NR): unspecified proportion of population has condition.

^c^ROB: Overall risk of bias assessment.

Arab et al. [30] conducted a cross-sectional analysis using 12,693 participants from 3 subsets of the National Health and Nutrition Examination Survey (NHANES): (1) NHANES III (1988–1994) age 20–59, (2) NHANES III age 60+, and 3) NHANES (1999–2002) age 60+ (Table 4). In subset 1, cognitive function domains were evaluated using subtests of the Neurobehavioral Evaluation System 2 (NES2): visuomotor speed (SRTT), information processing speed, concentration, and motor control (SDST), and the serial digit learning test (SDLT), which measures learning and recall (SDLT). In subset 2, cognitive testing evaluated attention and verbal memory (SRT), and in subset 3, attention, visuospatial skills, learning, and memory were assessed (DSST). Walnut consumption was characterized using a single 24-hour dietary recall as walnuts with high certainty (WWHC; tertiles from >0 to >11 g), walnuts with other nuts (WWON; tertiles from >0 to >7.7 g), or no reported nut consumption (NRNC). Among the WWHC group, higher walnut consumption was significantly associated with improved performance on all tests, after adjustment for covariates (*p*-trend < .01). Additionally, the effect size and significance levels were greater for the highest tertile of walnut consumption than for the middle and lower tertiles, demonstrating a dose-response effect. Similar findings were reported for the WWON group. However, risk of bias for this study was rated as some concerns, due to participant selection (non-random sampling and no description of compatibility between responders and non-responders) and exposure/outcome ascertainment (Table 6).

**Table 6.** ROB assessment of included observational studies using the Newcastle-Ottawa score for cohort and cross-sectional studies^a^.

Cross-sectional studies					
Study (Year)	Selection (maximum: ★★★★★)	Comparability (maximum: ★★)	Outcome (maximum: ★★★)	Score (out of 10)	Overall ROB
Arab et al. (2015) [30]	★★★★	★★	★★	8	SC
Arab et al. (2019) [29]	★★★★	★	★★	7	SC
Bishop et al. (2020) [31]	★★★★	★★	★★★	9	Low
Guasch-Ferré et al. (2017) [20]	★★	★	★★★	6	SC
Liu et al. (2020) [33]	★★	★	★★★	6	SC
Valls-Pedret et al. (2012) [32]	★★★★	★★	★	7	SC

Prospective Cohort Study					
Study (Year)	Selection (Maximum: ★★★★★)	Comparability (Maximum: ★★)	Outcome (Maximum: ★★★★)	Score (Out of 11)	Overall ROB
O’Brien et al. (2014) [34]	★★★	★★	★★	7	SC

ROB: risk of bias; SC: some concerns.

^a^Lower scores indicate increased risk; Cross-Sectional: 0–4 = High; 5–7 = SC; 8–10 = Low; Cohort: 0–4 = High; 5–7 = SC; 8–11 = Low.

Valls-Pedret et al. [32] conducted a cross-sectional baseline analysis using 447 older adults enrolled in the Prevención con Dieta Mediterránea study (PREDIMED; 2004–2009) at high risk for cardiovascular disease, including subjects with T2D, HLD, and HTN (Table 4). Cognitive tests were administered by a neuropsychologist blinded to subjects’ exposures to examine global cognitive function (MMSE), immediate and delayed episodic verbal memory (RAVLT), episodic memory of performance (VPAT), semantic fluency (animal fluency test), immediate and working memory (digit span-forward/backward [WAIS subtest]), and executive function (CTT-I, II). Walnut consumption was quantified by a face-to-face interview using a validated FFQ and characterized as a continuous regression coefficient of per 30 g/d increase in walnuts. Higher walnut consumption was significantly associated with improved performance only on the digit span-backward test of working memory (regression coefficient = 1.19, CI: 0.061, 2.322, *p* = .039), after adjustment for covariates. Overall risk of bias for this study was rated as some concerns, due to participant selection (non-random sampling and no description of compatibility between responders and non-responders) and incomplete reporting of results (significant findings only) (Table 6).

Bishop et al. [31] conducted a cross-sectional study of 3,632 older adults (age 65+, majority overweight or obese) enrolled in the Health and Retirement Study (HRS) and Health Care and Nutrition (HCN) Study from 2012 to 2016 (Table 4). Walnut consumption was characterized using a single FFQ in 2013, as no intake, low intake (0.01 − 0.08 oz/d, mean = 0.04 oz/d), or moderate intake (> 0.08 oz/d, mean = 0.49 oz/d), and g global cognitive function was evaluated using the Telephone Interview for Cognitive Status (TICS) in 2012, 2014, and 2016. Subjects reporting low or moderate walnut consumption had significantly higher TICS scores relative to subjects reporting no walnut consumption at all three time points (*p* < .001). However, latent growth models found no significant association between walnut consumption and change in global cognitive status (TICS scores) over four years, after adjustment for covariates. Risk of bias for this study was rated as low (Table 6).

In a population-based prospective cohort study, O’Brien et al. [34] examined 15,467 older women enrolled in the Nurses’ Health Study (1995–2001) (Table 4). The study population included various proportions of subjects with HTN, HLD, and T2D, and 52% were either overweight or obese. Walnut intake was classified by 28 g servings using a single validated FFQ as <1 serving/month, 1–3 servings/month, or ≥1 servings/week. The highest category of walnut consumption encompassed the original frequency categories of 1 serving/week, 2–4 servings/week, and ≥5 servings/week, which were collapsed due to low responses. 1 servings/week. The highest category of walnut consumption encompassed the original frequency categories of 1 serving/week, 2–4 servings/week, and ≥5 servings/week, which were collapsed due to low responses. Cognitive function was evaluated four times over the six-year study using three scores: (1) TICS score, (2) verbal memory score (average of EBMT [immediate, delayed recall], fluency test, delayed recall of word list of TICS, digit span-backward), and (3) global score (average of all 6 tests). Subjects who consumed 1–3 servings/month of walnuts had significantly higher global cognitive and verbal memory scores than those who consumed <1 serving/month, after adjustment for covariates (*p*-value not reported). The authors noted that these findings should be interpreted with caution, as the small sample size in the highest walnut consumption frequency category was not significantly associated with improved cognitive function. Furthermore, there was no overall trend of increasing walnut intake with improved cognitive performance (p-trends for TICS, global score, verbal memory: 0.66, 0.90, 0.51). Additionally, there were no significant differences between walnut intake groups in rates of cognitive decline over the six years of follow-up on any of the composite scores (p-trends for TICS, global score, verbal memory score: 0.42, 0.27, 0.29). Overall risk of bias was rated as some concerns, due to participant selection (specific group of subjects, i.e. nurses, and no exclusion of subjects with cognitive impairment at start), no description of subjects lost to follow-up, and selective reporting (Table 6).

### Stroke

#### Cohort & cross-sectional studies

Two cross-sectional studies [20,33] examining the association between walnut consumption and risk of fatal and non-fatal stroke were included (Table 4). Both analyses used data from three prospective cohort studies, and the same time periods from these studies: the Nurses’ Health Study (NHS, 1986–2012), the NHS II (1991–2013), and the Health Professionals Follow-Up Study (HPFS, 1986–2012). The total number of participants analyzed differed slightly between Guasch-Ferré et al. (210,836) and Liu et al. (192,655), possibly due to differences in inclusion/exclusion criteria (e.g. Liu et al. excluded participants with T2D at baseline). Subject populations were similar in gender proportions (19.6% or 17.7% male) and age (mean age of reported subgroups ranged from 43.2 to 64.4).

Guasch-Ferré et al. [20] found that walnut intake (≥1 serving/week) was associated with a 17% reduction in risk for stroke (HR: 0.83, 95% CI: 0.71–0.96), after adjustment for covariates (Table 4). Similarly, Liu et al. [33] found that increased walnut consumption (per 0.5 serving/day) over a 4-year interval was associated with a 20% lower risk of stroke (RR: 0.80, 95% CI: 0.67–0.95) during the subsequent 4-year interval, after adjustment for covariates. Risk of bias for both studies was rated as some concerns, due to participant selection (selected group of users, i.e. health professionals, and no description of compatibility between responders and non-responders), no adjustment for education level, and exposure ascertainment (Table 6).

### Mood

#### RCTs

Three randomized (2 cross-over [36,39] and 1 parallel [38]) trials reporting the effects of walnut on mood outcomes were included. All three studies were conducted in generally healthy young adults (mean age: 19.9–24) (Table 2). Walnut interventions were variable: a single dose of 30 mL walnut oil, a single dose of 41.6 g walnuts, or 60 g/d walnuts for 8 weeks. Despite heterogeneity in walnut interventions, findings across studies were consistent; no significant difference in mood was found between walnut and control interventions in the complete study populations.

Probst et al. [39] used the Multidimensional Mood State Questionnaire (MDMQ) to examine mental state (mood, fatigue, and calmness) in young healthy males after a single meal of yogurt with 41.6 g walnuts or no walnuts in a cross-over design. No significant difference in mood was detected between walnut and control interventions (Table 3). Risk of bias for this study was rated as high due to the randomization process and missing outcome data (Figure 2).

Miller et al. [38] also examined the acute effects of walnuts on mood in healthy young adults. A single dose of 30 mL walnut oil in a milkshake did not have a significant effect on total mood disturbance score (TMD), compared to a milkshake with no added fat, using the Profile of Mood States Questionnaire (POMS, 5 domains: tension-anxiety, depression-dejection, anger-hostility, vigor-activity, fatigue-inertia, confusion-bewilderment). Risk of bias for this study was rated as high, due to the randomization process and deviations from the intended intervention (Figure 2).

Pribis et al. [36] also used the POMS questionnaire to assess mood in healthy young adults over a longer study duration than the other two studies. Subjects consumed banana bread with either 60 g/d walnuts or no added walnuts for 8 weeks in a cross-over design. No significant difference in TMD or subdomains was observed between walnut and control interventions in the whole study population. However, in a subsequent analysis by sex, walnut consumption in males significantly improved TMD score (i.e. a lower score) compared to placebo, despite no differences among females (Table 3). Risk of bias for this study was rated as some concerns, due to missing data.

#### Cross-sectional studies

One observational study investigating the associations between walnut consumption and mood was included [29]. Arab et al. conducted a cross-sectional analysis of 26,656 adults (mean age: 46) from the National Health and Nutrition Examination Survey (NHANES) between 2005 and 2014 [29] (Table 4). Walnut consumption was characterized using a 24-hour dietary recall as walnuts with high certainty (WWHC; mean 24 g/day), walnuts with other nuts (WWON; mean 4 g/day), or no walnuts (other nuts [ON], or no nuts [NN]). Depression scores for symptoms within the prior 2 weeks were based on PHQ-9 questionnaire responses. Walnut intake (WWHC and WWON) was significantly associated with lower total depression scores, after adjustment for covariates, and this effect was strongest for WWHC. Specifically, the least squared mean for total depression scores among WWHC consumers was 26% lower than NN subjects and 17% lower than ON consumers. Among the individual PHQ-9 subdomains, walnut consumption was significantly associated with increased concentration, energy levels, interest in doing things, self-control of rates of speech, and movement, and reduced hopelessness relative to NN consumption. The observed inverse associations between walnut intake and depression scores were influenced by sex as greater effects were observed among women (32% reduction, *p* < .0001) compared to men (21% reduction, *p* = .05), possibly due to increased likelihood of reporting depression symptoms. Risk of bias for this study was rated as some concerns, due to participant selection, lack of adjustment for important covariates (e.g. education), and exposure/outcome ascertainment (Table 6).

### Outcomes related to risk factors for cognitive decline

#### Glucose homeostasis

##### Haemoglobin A1C (HbA1C)

**Figure 3.**
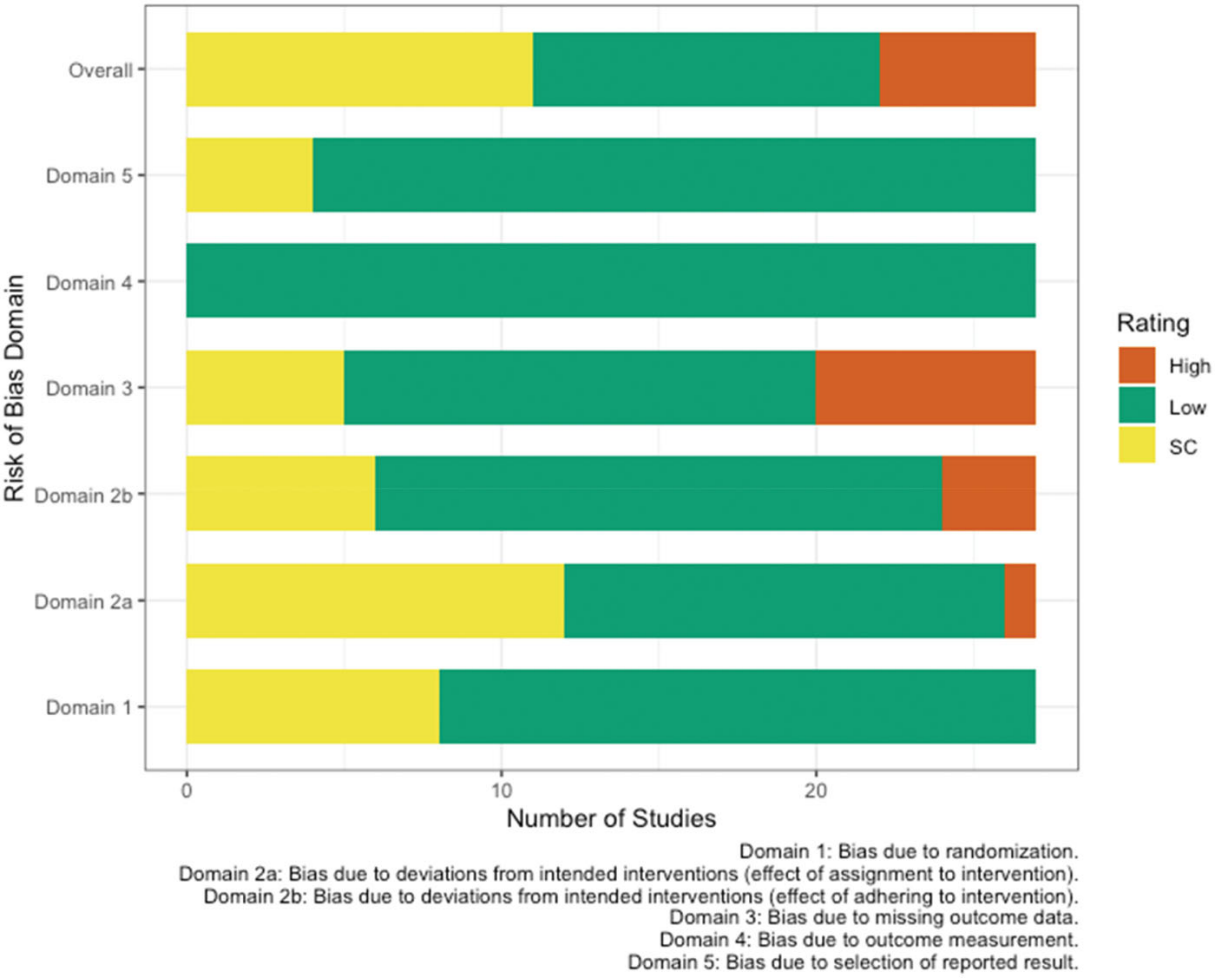
Risk of bias assessment based on the *Cochrane risk-of-bias tool for randomized trials (RoB 2)* conducted for twenty-seven publications reporting glucose homeostasis and inflammatory outcomes [HbA1c, HOMA-IR, hsCRP, TNF-a, IL-6, IL-1β, E-selectin, sVCAM, sICAM]. SC; some concerns.

Ten randomized (5 cross-over [40–44] and 5 parallel [45–49]) trials reporting HbA1c were included. Of these, one study was conducted in overweight or obese subjects [47], five were in subjects at risk for [43] or with T2D [42,45,46,49], two were in subjects with metabolic syndrome (MetS) [41,48], and two were in generally healthy individuals [40,44]. Walnut intervention doses ranged from 15 to 56 g/day of walnuts and/or walnut oil and intervention durations ranged from 8 weeks to 14 months (Table 5). Overall, risk of bias was rated as some concerns, primarily due to deviations from the intended intervention and missing outcome data (Figure 3; Additional information can be found in Supplemental Table S2).

**Table 5.** Study characteristics of RCTs reporting risk factors for cognitive-decline^a^.

Study (Year)	Country (Funding)	Walnut Intervention	Control Intervention^b^	RCT Design	Baseline Health (%)^c^	Mean BMI (SD)	N Randomized (N Analysed)	Mean Age (SD), Years	% Male	Washout Period	Total Duration (Exposure Duration)	Risk Factor Outcome(s)	ROB^d^
Aronis et al. (2012) [50]	USA (Aca)	48 g/d walnuts (liquid meal)	Isocaloric liquid meal	RCO	MetS (100): Obese (100), HTN (NR), HLD (NR)	36.6 (1.7)	15 (15)	58 (2.5)	60	1 mo	8 wks (4d)	hsCRP, sICAM-1, sVCAM-1, E-selectin, TNFɑ, IL-6	Low
Bamberger et al. (2017) [40]	Germany (Ind)	43 g/d shelled walnuts	Nut-free diet	RCO	Healthy (100), Overweight/Obese (NR), CVD (0)	25.1 (4.0)	204 (194)	63 (7)	30.9	4 wks	24 wks (16 wks)	HbA1C	High
Bhardwaj et al. (2018) [51]	USA (NR)	60 g shelled walnuts in single high-fat (62.3% energy) meal	77 g almonds in a single high-fat (62.3% energy) meal	RCO	Overweight (100), T2D (0), CVD (0), HTN (0)	28.6 (2.2)	27 (27)	42.3 (6.8)	40.7	2 wks	2 wks (4 hrs)	sVCAM-1	Low
Burns-Whitmore et al. (2014) [52]	USA (Gov, Ind)	28.4 g walnuts, 6x/wk	Standard egg, 6x/wk	RCO	Healthy (100)	23 (1.0)	26 (20)	38 (NR)	20	4 wks	32 wks (8 wks)	TNFɑ, E-selectin, sICAM-1, hsCRP, IL-6	High
Canales et al. (2011) [53]	Spain (Aca)	21.4 g/d walnuts in steak and sausage (walnut-enriched meat)	Low-fat meat without walnuts	RCO	*Increased CVD risk:* Overweight (60), Obese (40), HTN (NR), HLD (NR)	NR	25 (22)	54.8 (NR)	60	4–6 wks	14–16 wks (5 wks)	sVCAM-1, sICAM-1	SC
Chiang et al. (2012) [54]	USA (Ind)	42.5 g walnuts, 6x/wk	No walnuts or fish	RCO	HLD (NR)	24.8 (NR)	27 (25)	33 (NR)	56	1 d	12 wks (4 wks)	hsCRP, sICAM-1, sE-selectin, TNFɑ, IL-6, IL-1Β	High
Cortes et al. (2006) [55]	USA (Gov, Aca)	42 g/d walnuts in high-fat (63% energy) meal	25 g olive oil in high-fat (63% energy) meal	RCO	HLD (50), Overweight (NR), HTN (0)	25.5 (3.3)	24 (24)	38.5 (10.5)	83.3	1 wk	4 hrs	sICAM-1, sVCAM-1, E-selectin	Low
Damasceno et al. (2011)[56]	Spain (Ind)	40–65 g/d walnuts on Mediterranean-type diet	Isoenergenic Mediterranean-type diet with 35–50 g/d virgin olive oil	RCO	HLD (100)	25.7 (2.3)	20 (18)	56 (13)	50	None	12 wks (4 wks)	sICAM-1, sVCAM-1, hsCRP	SC
Fatahi et al. (2019) [57]	Iran (Gov)	9 walnut*s* + 150 g fatty fish/wk	300 g fatty fish/wk	RP	Overweight/Obese (100), T2D (0), HTN (0), CVD (0)	33.3 (5.6)	99 (99)	53.4 (1.6)	0	NA	12 wks (12 wks)	hsCRP, Il-6, TNFɑ	Low
Holscher et al. [58]	USA (Gov, Ind)	42 g/d walnuts	No walnuts (No active intervention)	RCO	Healthy (100)	28.8 (0.9)	18 (18)	53.1 (2.2)	55.6	1 wk	7 wks (3 wks)	sICAM-1, sVCAM-1	Low
Hwang et al. (2019) [41]	Korea (Ind)	45 g/d walnuts	Isocaloric white bread	RCO	MetS (100): Overweight/Obese (NR), HTN (NR), HLD (NR)	27.1 (2.6)	119 (84)	39.5 (14.5)	50	6 wks	38 wks (16 wks)	hsCRP, HbA1C	SC
Katz et al. (2012) [59]	USA (Ind)	56 g/d shelled, unroasted walnuts (walnut-enriched ad libitum diet)	Ad libitum diet without walnuts	RCO	MetS (100): Overweight/Obese (NR), HTN (NR), HLD (NR)	33.2 (4.4)	46 (40)	57.4 (11.9)	39.1	4 wks	24 wks (8 wks)	HOMA-IR	High
Ma et al. (2010)[42]	USA (NR)	56 g/d shelled, unroasted walnuts (walnut-enriched ad libitum diet)	Ad libitum diet without walnuts	RCO	T2D (100), Overweight/Obese (NR), HTN (NR), HLD (NR), CVD (0)	32.5 (5.0)	24 (21)	58 (9.2)	41.7	8 wks	28 wks (8 wks)	HbA1C, HOMA-IR	SC
Pieters et al. (2005) [60]; Mukuddem-Petersen et al. (2007)[61]	South Africa (Gov, Ind)	63–108 g/d walnuts (20% energy)	Nut-free diet	RP	MetS (100): Obese (91), HTN (NR), HLD (NR)	35.2 (NR)	68 (64)	45 (10)	45.3	NA	11 wks (8 wks)	HOMA-IR; hsCRP	Low
Nijke et al. [43]	USA (Ind)	56 g/d walnuts on calorie-adjusted diet	Calorie adjusted diet without walnuts	RP	*Increased risk for T2D*: Overweight (NR), HLD (NR), HTN (NR), T2D (0), CVD (0)	30.1 (4.1)	112 (97)	54.9 (11.4)	30.4	12 wks	15 mo (6 mo)	HbA1C	SC
Rock et al. (2016) [62]	USA (Gov, Ind)	42 g/d walnuts (higher fat [35% energy], lower CHO [45% energy] diet)	No walnuts (Higher fat [35% energy], lower CHO [45% energy] diet	RP	Overweight/Obese (100), Insulin resistant (51.4), T2D (0)	33.5 (NR)	245 (194)	50 (NR)	0	NA	1 yr (1 yr)	hsCRP, IL-6, HOMA-IR	SC
Ros et al. (2004)[63]	Spain (Gov, Ind)	40–65 g/d walnuts	Isoenergetic Mediterranean-type diet without walnuts	RCO	HLD (100)	NR	21 (20)	55 (NR)	40	None	8 wks (4 wks)	sICAM-1, sVCAM-1, hsCRP	Low
Tapsell et al. (2004) [46]	Australia (Ind)	30 g/d walnuts (low-fat/modified-fat diet)	No walnuts (low-fat/modified-fat diet)	RP	T2D (100)	29.2 (2.6)	58 (55)	59.3 (8.1)	56	NA	6 mo (6 mo)	HbA1C	SC
Tapsell et al. (2009) [45]	Australia (Ind)	30 g/d walnuts (low fat [30%] diet)	No walnuts (low fat [30%] diet)	RP	Overweight (100), T2D (100)	33.2 (4.2)	50 (35)	54 (8.7)	NR	NA	1 yr (1 yr)	HbA1C, HOMA-IR	Low
Tapsell et al. (2017) [47]	Australia (Ind, Aca)	30 g/d walnuts (+ Interdisciplinary Advice; IW)	No walnuts (Interdisciplinary Advice; I)	RP	MetS (34.9), Overweight/Obese (100), HTN (NR), HLD (NR)	32 (4.5)	377 (178)	44.3 (10.5)	26	NA	1 yr (1 yr)	HbA1C	Low
Tindall et al. (2019) [64]	USA (Gov, Aca, Ind)	57–99 g/d whole walnuts (WD)	Walnut fatty acid-matched diet (WFMD): same fatty acid composition as WD, but devoid of walnuts	RCO	*Increased risk for CVD:* Overweight/ Obese (100), HTN (NR), HLD (NR), T2D (0)	30 (1.0)	45 (36)	43 (10)	55.6	23 d	186 d (6 wks)	hsCRP	SC
Wu et al. (2010) [48]	China (Gov, Aca, Ind)	30 g/d walnuts (in isocaloric bread)	30 g/d flaxseed (in isocaloric bread)	RP	MetS (63.4), HTN (61.8), Overweight/Obese (NR), HLD (NR), CVD (0)	25.4 (2.5)	283 (277)	48.4 (NR)	55.8	NA	12 wks (12 wks)	HbA1C	Low
Wu et al. (2014) [44]	Germany (Ind)	43 g/d shelled walnuts	No walnuts (Isocaloric background diet)	RCO	Healthy (100), Obese (0), HLD (0), HTN (0), T2D (0)	24.9 (0.6)	57 (40)	60 (1)	25	2 wks	18 wks (8 wks)	HbA1C. HOMA-IR, sVCAM-1, sICAM-1	SC
Zhao et al. (2004, 2007) [65,66];	USA (Ind)	37 g/d walnut and 15 g/d walnut oil (ALA diet)	No walnuts (AAD diet)	RCO	HLD (100), Overweight/Obese (100), T2D (0)	28.1 (0.7)	23 (23)	49.8 (1.6)	86.9	3 wks	27 wks (18 wks)	sICAM-1, sVCAM-1, hsCRP, E-selectin; TNFɑ, IL-1β, IL-6	SC
Zibaeenezhad et al. (2016) [49]	Iran (Aca)	15 g/d walnut oil	No walnuts (No active intervention)	RP	T2D (100), Overweight/Obese (NR)	27.4 (2.4)	100 (100)	54.8 (11.1)	47.8	NA	3 mo (3 mo)	HbA1C	SC

^a^AAD: Average American Diet; Aca: Academia; ALA: alpha-linoleic acid; BMI: Body Mass Index (kg/m^2^); CHO: carbohydrate; CVD: cardiovascular disease (includes coronary artery disease, myocardial infarction, peripheral vascular disease, congestive heart failure, or carotid stenosis); Gov: Government; HbA1C: haemoglobin A1C; HLD: hyperlipidaemia (includes hypercholesterolemia); HOMA-IR: homeostatic model assessment-insulin resistance; hsCRP: high sensitivity c-reactive protein; HTN: hypertension; IL: interleukin; Ind: Industry; MetS: metabolic syndrome (includes proportion of HTN, HLD, overweight/obese); *N*: number; NA: not applicable; NR: not reported; RCO: randomized cross-over; RCT: randomized-controlled trial; ROB: risk of bias; RP: randomized parallel; SC: some concerns; SD: standard deviation; sICAM-1: soluble intracellular adhesion molecule-1; sVCAM-1: soluble vascular cell adhesion molecule-1; T2D: type-II diabetes; TNFɑ: tumour necrosis factor-alpha; USA: United States of America.

^b^For studies with multiple walnut-free intervention arms, the intervention most similar to the walnut intervention was chosen as the control group.

^c‘^Healthy’: no chronic illness (e.g. T2D, CVD, MetS, etc.); ‘Health condition (NR)’: unspecified proportion of population has condition.

^d^ROB: Overall risk of bias assessment.

**Figure 4.**
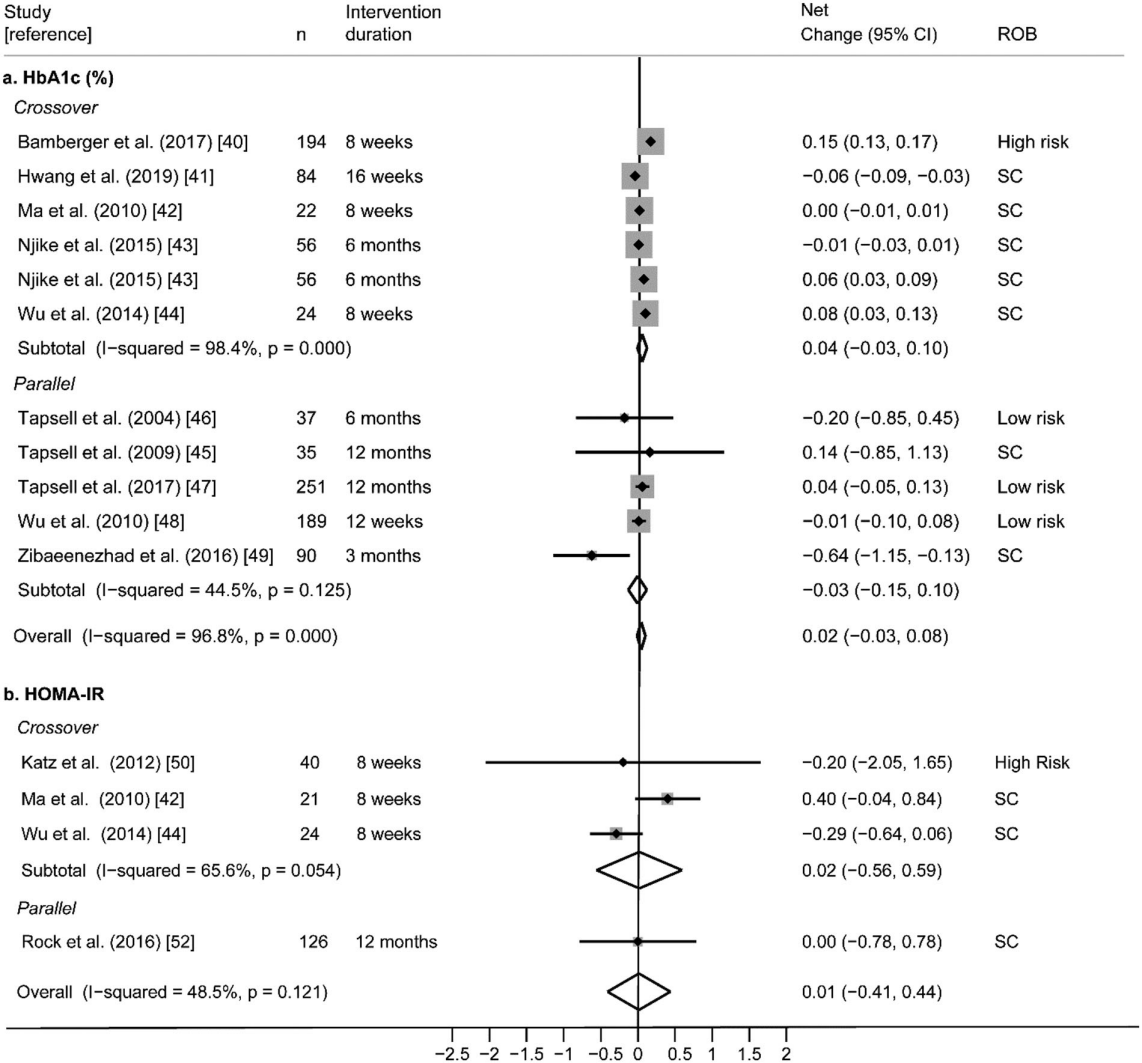
(a) Effect of walnut intake on HbA1c, reported in ten RCTs with plausible data. (b) Effect of walnut intake on HOMA-IR, reported in 4 RCTs with plausible data. Weights are derived from random-effects analysis. Each grey box represents the individual study’s effect estimate, and the horizontal line represents the 95% CI of the effect estimate. The diamond shape represents the meta-analysis pooled effect estimate and its CI. A vertical line displays the location of the meta-analysis pooled effect estimate. n: number of participants; CI: confidence interval; ROB: risk of bias; SC: some concerns.

Findings were relatively consistent; seven of the ten studies did not find a significant difference in HbA1c between control and walnut interventions. Two studies found that walnut significantly decreased HbA1c [41,49], and one study found that walnut significantly increased HbA1c [40]. However, random-effects meta-analysis of these ten trials did not find significant overall effects of walnut on HbA1c (pooled net change = 0.02%; 95% CI −0.03%, 0.08%) with high statistical heterogeneity (I^2^ = 96.8%; *p* < .0001) (Figure 4).

##### Homeostatic model assessment-insulin resistance (HOMA-IR)

Six randomized (3 cross-over [42,44,59] and 3 parallel [45,60,62]) trials reporting HOMA-IR were included. Of these, four studies were conducted in subjects with T2D and/or MetS [42,45,59,60], one was in overweight or obese subjects [62], and one was in generally healthy subjects [44]. Walnut interventions using doses ranging from 30 to 56 g/day were compared to controls and intervention durations ranged from 8 weeks to 12 months (Table 5). Overall, risk of bias was rated as some concerns, primarily due to missing outcome data and deviations from the intended interventions (Figure 3).

Findings within these six individual RCTs were consistent; none found a significant difference in HOMA-IR between walnut and control interventions. Random-effects meta-analysis of the four RCTs reporting quantitative HOMA-IR data (Figure 4) did not find significant effects of walnut on HOMA-IR (pooled net change = 0.01%; 95% CI −0.41%, 0.44%) with moderate statistical heterogeneity (I^2^ = 48.5%; *p* = 0.121). Findings from the two studies that could not be meta-analysed were consistent with the meta-analysis as no significant difference in HOMA-IR was found between walnut and control interventions [45,60] (Supplemental File 2).

#### Inflammation

##### High sensitivity c-reactive protein (hsCRP)

Eleven randomized (8 cross-over [40,41,52,54,56,63–65] and 3 parallel [57,61,62]) trials reporting hsCRP as an outcome were included. Of these, four studies were conducted in subjects with HLD [54,56,63,65], two were in subjects with MetS [41,61], three were in subjects who were overweight or obese [57,62,64], and two were in generally healthy participants [40,52]. Walnut interventions with doses ranging from 28.4 to 99 g/day were compared to controls and intervention durations ranged from 4 weeks to 12 months (Table 5). Overall, risk of bias was rated as some concerns, primarily due to issues arising with the randomization process and missing outcome data (Figure 3).

**Figure 5.**
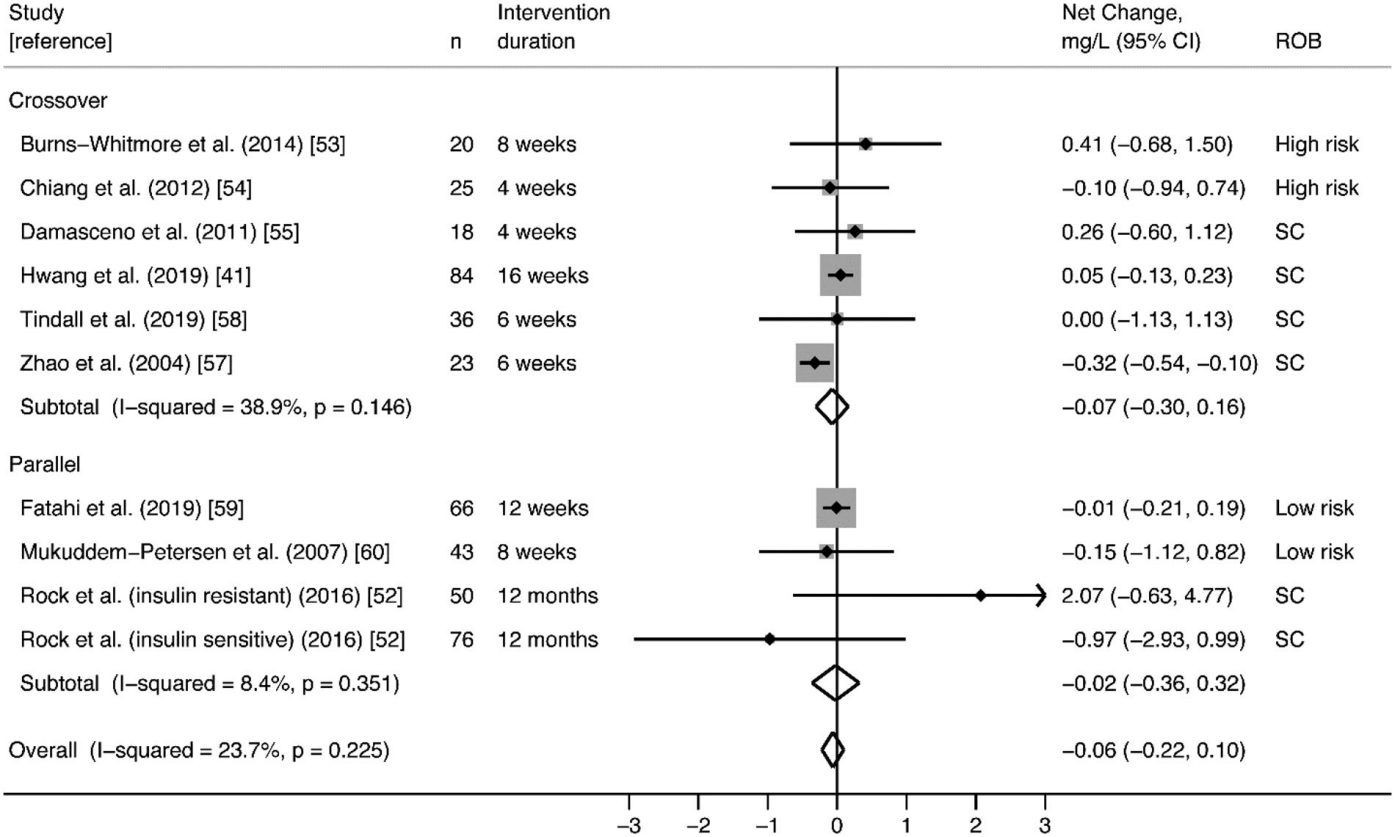
Effect of walnut intake on hsCRP, reported in nine RCTs with plausible data. Weights are derived from random-effects analysis. Each grey box represents the individual study’s effect estimate, and the horizontal line represents the 95% CI of the effect estimate. The diamond shape represents the meta-analysis pooled effect estimate and its CI. A vertical line displays the location of the meta-analysis pooled effect estimate. n: number of participants; CI: confidence interval; ROB: risk of bias; SC: some concerns.

Findings were consistent; ten studies did not observe significant effects of walnut on hsCRP, and one study reported a significant reduction in hsCRP with walnut consumption [65]. Random-effects meta-analysis of nine RCTs reporting plausible data did not find significant effects on hsCRP (pooled net change = −0.06 mg/L; 95% CI −0.21 mg/L, 0.09 mg/L) with low statistical heterogeneity (I^2^ = 20.3%; *p* = .251) (Figure 5). In the two studies that could not be meta-analysed [40,63], no significant differences in hsCRP were observed between walnut and control interventions (Supplemental File 2).

##### Soluble intracellular adhesion molecule-1 (sICAM-1)

Ten randomized cross-over trials reported sICAM-1 as an outcome [44,50,52–56,58,63,65]. Of these, one study was conducted in obese subjects with MetS [50], five were in subjects with HLD [54–56,63,65], one was in subjects with mixed cardiovascular disease risk factors [53], and three were in generally healthy subjects [44,52,58]. Various walnut interventions using doses ranging from 21.4 to 65 g/day were compared to controls and intervention durations ranged from 4 days to 8 weeks (Table 5). Overall, risk of bias across these studies was variable; four studies were rated as low [50,55,58,63], four studies as some concerns [44,53,56,65], and two as high [52,54] (Figure 3).

**Figure 6.**
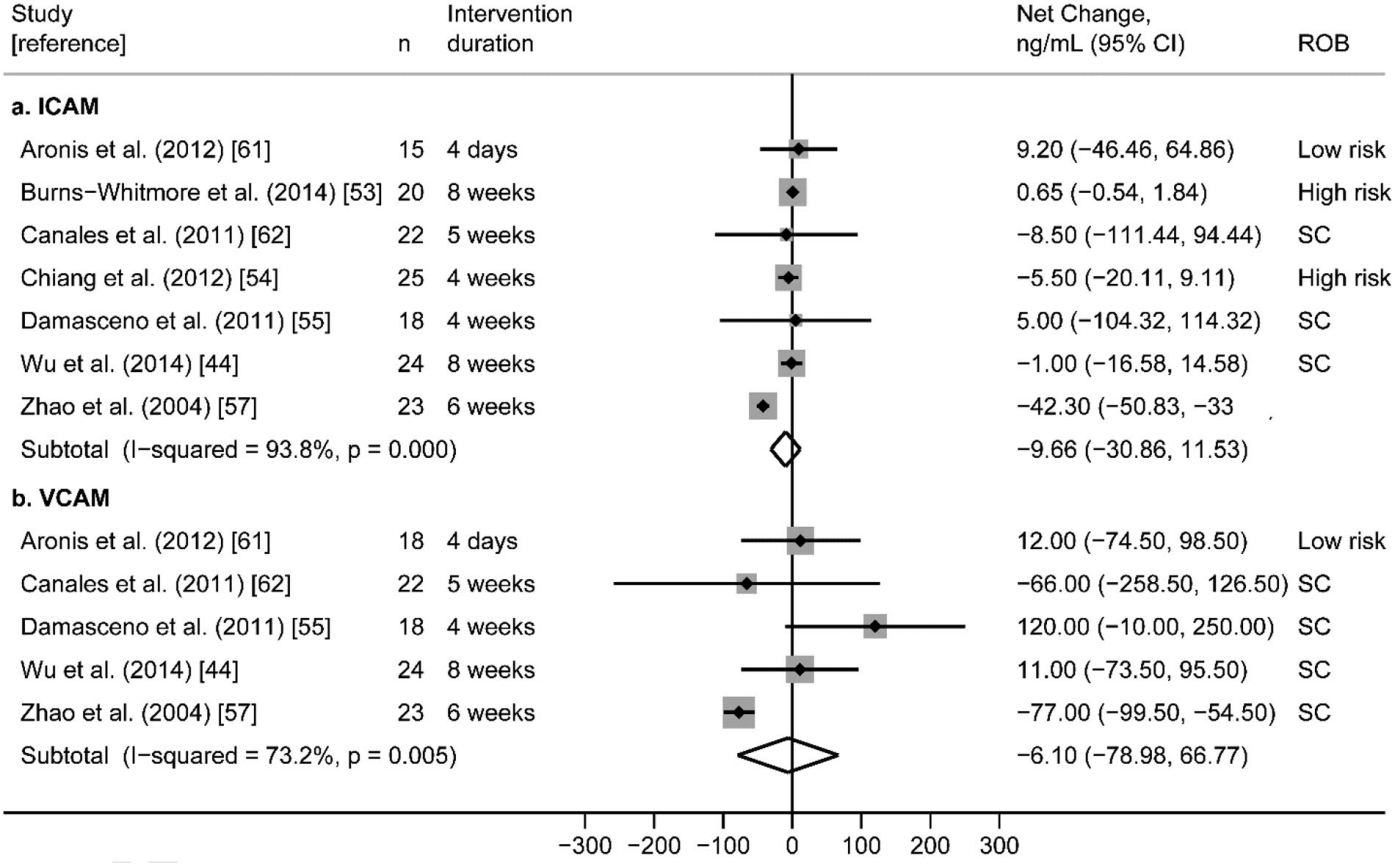
**(**a) Effect of walnut intake on sICAM-1, reported in seven crossover RCTs with plausible data. (b) Effect of walnut intake on sVCAM-1, reported in five crossover RCTs with plausible data. Weights are derived from random-effects analysis. Each grey box represents the individual study’s effect estimate, and the horizontal line represents the 95% CI of the effect estimate. The diamond shape represents the meta-analysis pooled effect estimate and its CI. A vertical line displays the location of the meta-analysis pooled effect estimate. *n*: number of participants; CI: confidence interval; ROB: risk of bias; SC: some concerns.

Findings were relatively consistent across studies; nine of the ten RCTs found no significant differences in sICAM-1 between walnut and control interventions. Only one study found that the walnut intervention significantly reduced sICAM-1 compared to control. [65] Random-effects meta-analysis of seven RCTs reporting sICAM-1 plausible data (Figure 6) did not find significant effects on sICAM-1 (pooled net change = −9.66 ng/mL; 95% CI −30.87 ng/mL, 11.54 ng/mL) with high statistical heterogeneity (I^2^ = 93.8%; *p* < 0.0001). Findings from the three studies that could not be meta-analysed [55,58,63] were consistent with the meta-analysis as no significant difference in sICAM-1 was found between walnut and control groups (Supplemental File 2).

##### Soluble vascular cell adhesion molecule-1 (sVCAM-1)

Nine randomized cross-over trials reported sVCAM-1 as an outcome [44,50,51,53,55,56,58,63,65]. Of these, one study was conducted in obese subjects with MetS [50], four were in subjects with HLD [55,56,63,65], one study was in overweight subjects [51], one was in subjects with mixed cardiovascular disease risk factors [53], and two were in healthy subjects [44,58]. Various walnut interventions using doses ranging from 21.4 to 60 g/day were compared to controls and intervention durations ranged from 4 days to 8 weeks (Table 5). Overall, risk of bias across studies was variable; five studies were rated as low [50,51,55,58,63], and four studies were rated as some concerns [44,53,56,65] (Figure 3).

Findings were inconsistent; two RCTs found that the walnut intervention significantly reduced sVCAM-1 relative to control [63,65], while the remaining seven studies found no significant difference in sVCAM-1 between walnut and control groups. Random-effects meta-analysis of the five RCTs reporting analysable sVCAM-1 data (Figure 6) did not find significant effects on sVCAM-1 (pooled net change = −6.18 ng/mL; 95% CI −79.36 ng/mL, 66.99 ng/mL) with high statistical heterogeneity (I^2^ = 73.2%; *p* = .005). Findings from three of the four studies that could not be meta-analysed were consistent with the meta-analysis; no significant difference in sVCAM-1 was found between walnut and control groups [51,55,58]. However, Ros et al. found that the walnut intervention significantly lowered sVCAM-1 relative to the control group (*p* = .045) [63] (Supplemental File 2).

##### Interleukin-6 (IL-6)

Six randomized (4 cross-over [50,52,54,66] and 2 parallel [57,62]) trials reporting IL-6 were included. Of these, one study was conducted in obese subjects with MetS [50], two were in obese or overweight subjects [57,62], two were in subjects with HLD [54,66], and one was in generally healthy subjects [52]. Walnut interventions using doses from 24.8 to 48 g/day were compared to controls and the intervention durations ranged from 4 days to 12 months (Table 5). Overall, risk of bias was variable; two studies were rated as low [50,57], two were rated as some concerns [62,66], and two were rated as high [52,54] (Figure 3).

**Figure 7.**
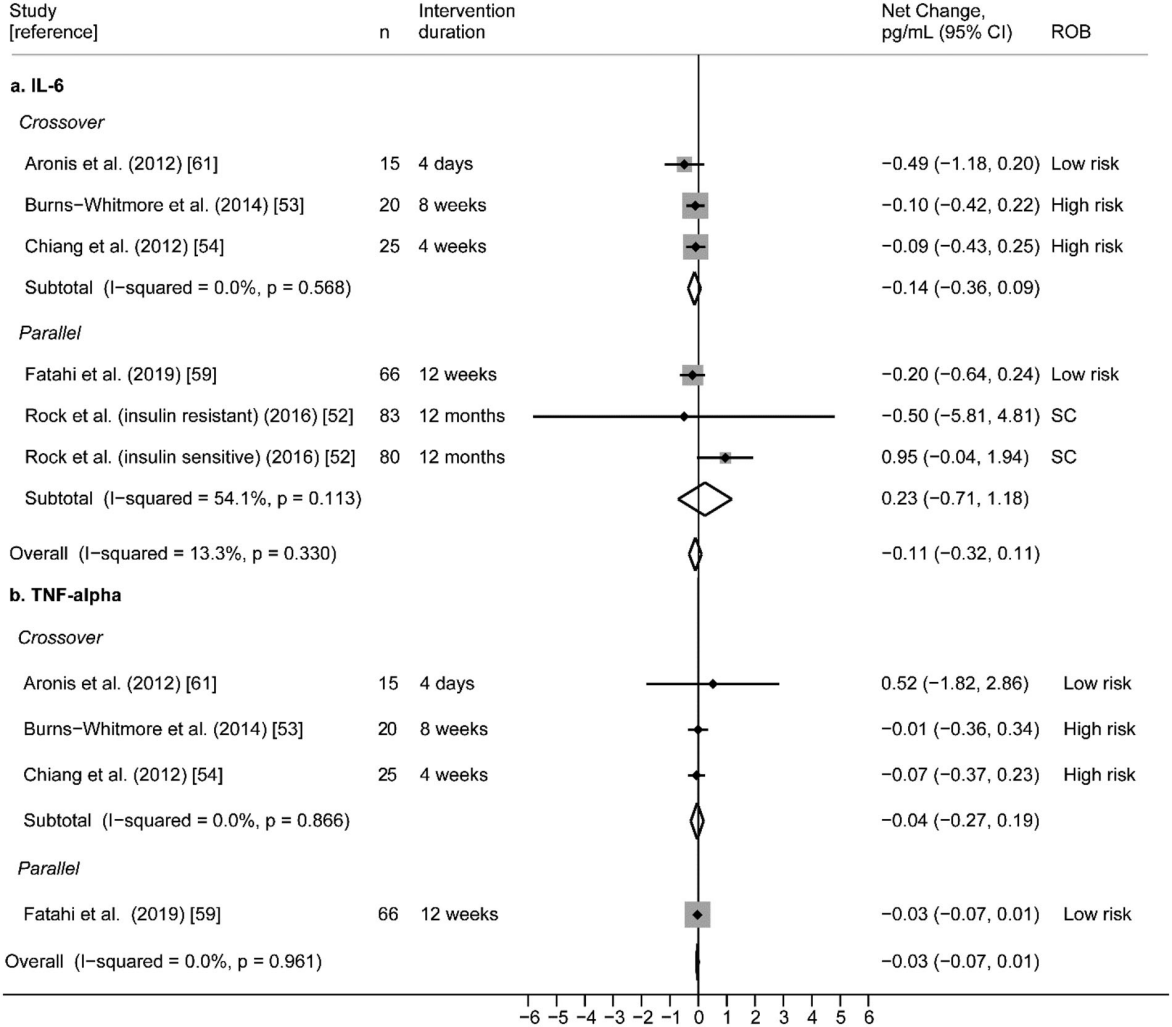
(a) Effect of walnut intake on IL-6, reported in five RCTs with plausible data. (b) Effect of walnut intake on TNF-α, reported in four RCTs with plausible data. Weights are derived from random-effects analysis. Weights are derived from random-effects analysis. Each grey box represents the individual study’s effect estimate, and the horizontal line represents the 95% CI of the effect estimate. The diamond shape represents the meta-analysis pooled effect estimate and its CI. A vertical line displays the location of the meta-analysis pooled effect estimate. n: number of participants; CI: confidence interval; ROB: risk of bias; SC: some concerns.

None of the six RCTs found a significant difference in IL-6 between walnut and control interventions. Random-effects meta-analysis of the five RCTs reporting plausible IL-6 data (Figure 7) did not find significant effects of walnut on IL-6 (pooled net change = −0.11 pg/mL; 95% CI −0.71 pg/mL, 1.18 pg/mL) with moderate statistical heterogeneity (I^2^ = 54.1%; *p* = .113). Findings from the one study that could not be meta-analysed was consistent with the results of the meta-analysis; no significant difference in IL-6 was found between the walnut and control intervention [66] (Supplemental File 2).

##### Tumour necrosis factor-alpha (TNFα)

Five randomized (4 cross-over [50,52,54,66] and 1 parallel [57]) trials reporting TNFα as an outcome were included. Of these, one study was conducted in subjects who were overweight or obese [57], one was in subjects with MetS [50], two were in subjects with HLD [54,66], and one study was in generally healthy subjects [52]. Various walnut interventions with doses ranging from 28.4 to 48 g/day were compared to controls, and intervention durations ranged from 4 days to 12 weeks (Table 5). Overall risk of bias was polarizing, with low [50,57] or high [52,54] ratings in equal distribution, and one study rated as some concerns [66] (Figure 3).

Overall, findings were consistent; four studies did not observe significant effects of walnut on TNFα [50,52,54,57]. Random-effects meta-analysis of these four RCTs reporting plausible data did not find significant effects of walnut on TNFα (pooled net change = −0.03 pg/mL; 95% CI −0.07 pg/mL, 0.01 pg/mL) with no statistical heterogeneity (I^2^ = 0.0%, *p* = .961) (Figure 7). One study [66] could not be meta-analysed; Zhao et al. [65] reported lower serum TNFα in subjects who consumed the walnut/alpha-linoleic acid diet (ALA diet) compared to the control diet (Supplemental File 2).

##### E-selectin

Five randomized cross-over trials reporting E-selectin as an outcome were included [50,52,54,55,65]. Of these, two studies were conducted in healthy subjects [52,55], one study was in subjects with MetS [50], and three were in subjects with HLD [54,55,65]. Varying walnut interventions with doses ranging from 28.4 to 48 g/day were compared to controls, with intervention durations ranging from 4 days to 8 weeks (Table 5). Overall risk of bias was polarizing, with low [50,55] or high [52,54] ratings in equal distribution, and one study rated as some concerns [65] (Figure 3). Findings from these five trials were inconsistent; in two RCTs, walnut significantly reduced E-selectin relative to the control [55,65], and no significant effects of walnut on E-selectin were observed in the other three studies.

**Figure 8.**
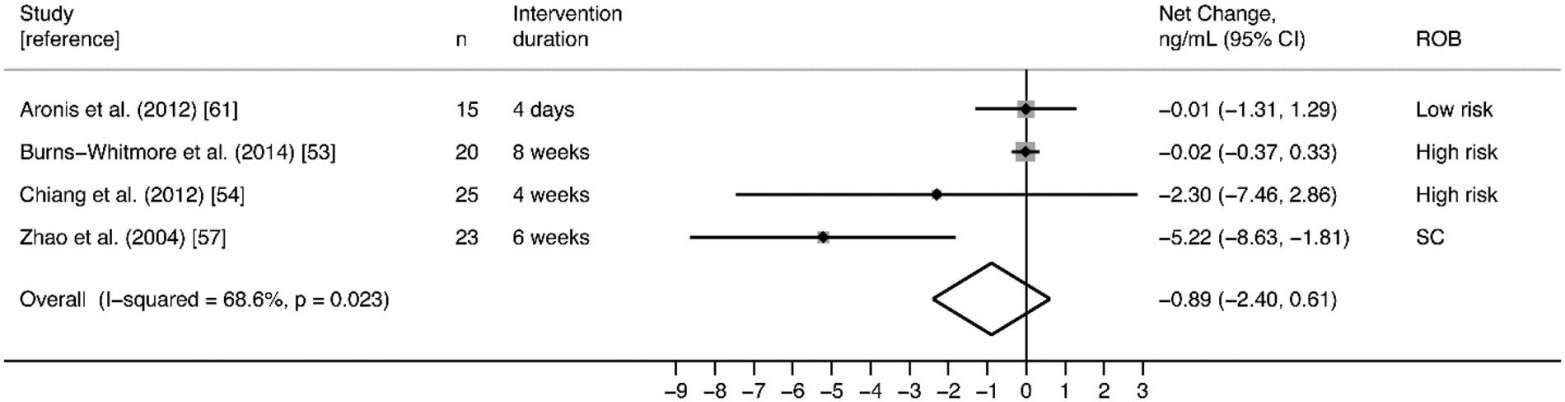
Effect of walnut intake on E-selectin, reported in four RCTs with plausible data. Weights are derived from random-effects analysis. Each grey box represents the individual study’s effect estimate, and the horizontal line represents the 95% CI of the effect estimate. The diamond shape represents the meta-analysis pooled effect estimate and its CI. A vertical line displays the location of the meta-analysis pooled effect estimate. n: number of participants; CI: confidence interval; ROB: risk of bias; SC: some concerns.

Random-effects meta-analysis of four RCTs reporting plausible data did not find significant effects on E-selectin (pooled net change = −0.89 ng/mL; 95% CI −2.40 ng/mL, 0.61 ng/mL) with moderate statistical heterogeneity (I^2^ = 68.6%; *p* = .023) (Figure 8). Only one study [55] with E-selectin as an outcome could not be meta-analysed; Cortes et al. reported a significant decrease in soluble E-selectin after the walnut meal, rather than the control meal (*p* = .033) (Supplemental File 2).

##### Interleukin-1 beta (IL-1β)

Only two RCTs (cross-over) [54,66] reported IL-1β, and therefore we did not conduct a meta-analysis on this outcome. Both studies included subjects with HLD, and in one study [66], all subjects were either overweight or obese. Walnut interventions were 37 g/day of walnuts with 15 g/day of walnut oil for 6 weeks [66] or 42.5 g/day of walnuts for 4 weeks [54] (Table 5). Findings across these studies were consistent; no differences in IL-1β were observed between walnut and control interventions. Overall risk of bias was rated as high [54] or some concerns [66], with variability in reported assay measures [66] and insufficient time between intervention periods for carryover effects to diminish [54] as rationale for overall judgement (Figure 3).

#### Strength of evidence rating

An evidence level of *low* was assigned for the effect of walnut consumption on cognitive function and mood in RCTs (i.e. further research is highly likely to have an important impact on our confidence in the estimate of association and is likely to change the estimate) (Table 7). An evidence level of *low* was also assigned for the association between higher walnut consumption and improved cognitive function in observational studies, with inconclusive effects on mood and stroke due to insufficient data. Regarding glucose homeostasis, an evidence level of *moderate* was assigned for no effect of walnut on HOMA-IR or HbA1C (i.e. further research is likely to have an important impact on our confidence in the estimate of association and may change the estimate). An evidence level of *moderate* was also assigned for no effect of walnut on inflammation outcomes.

**Table 7.** GRADE evidence profile table^a^.

Quality assessment							Strength of Evidence^b^	Summary & Justification
No of studies	Design (Ref.)	Limitations	Inconsistency	Indirectness	Imprecision	Dose-response		
**Cognition-related outcomes: Cognitive function, Mood, Stroke**								
5	RCTs [35–39]	Serious limitations: Overall ROB was high for 60% of trials and SC for 40% of trials. Studies were limited mostly due to concerns arising from the randomization process and de*via*tions from intended interventions.	No serious inconsistency: No trials reported significant differences in mood and total cognitive scores between walnut and control groups. Significant differences were observed in population subgroups and/or subdomains only.	No serious indirectness: Clinical and/or validated cognition-related tests used.	Imprecise: All studies reported wide CIs or other measures of variance.	Not applicable: no within study comparisons of different walnut intake amounts.	⊕⊕OO LOW	None of the 5 RCTs found a significant effect of walnut on mood or cognitive function in complete study populations, though subanalyses and/or subdomains demonstrated a walnut effect. Due to concerns regarding risk of bias and imprecision in measures, we conclude that the SOE for the effects of walnut intake on cognition-related outcomes is *low*.
7	Observational [20,29–34]	Some limitations: Overall ROB was SC for 86% of trials and low for 14% of trials. Studies were limited mostly due to participant selection, self-reported walnut intake, and incomplete or selective reporting of results.	No serious inconsistency: Significant differences were reported between walnut and control groups for mood (100%) and total cognitive function scores (80%). The remaining study reported a significant effect of walnut on a subdomain of cognitive function.	No serious indirectness: Clinical and/or validated cognition-related tests used.	Some imprecision: 43% of studies reported wide CIs and/or small sample sizes.	Dose-response is present for all cognition- related outcomes.	⊕⊕OO LOW	There are insufficient data to support a hypothesis on the associations between walnut intake and mood or stroke, a*s* ≤ 2 studies reported on these outcomes. However, 3 cross-sectional studies and 1 prospective cohort study found significant associations between walnut intake and cognitive function. Despite moderate imprecision and concerns of bias across these studies, the demonstration of a dose response effect upgraded the SOE for the association between walnut intake and cognition-related outcomes to a *low* rating.
**Inflammation**								
17	RCTs [41,44, 50–58,61–66]	Some limitations: Overall ROB was low for 44%, SC for 44%, and high for 12% of trials.	No serious inconsistency: Across studies reporting inflammation markers, 60–100% did not find a significant effect of walnut compared to control.	No serious indirectness: Biomarkers of inflammation.	Some imprecision: Results were imprecise across studies, as indicated by moderate or high statistical heterogeneity of meta-analyses.	Not applicable; no within study comparisons of different walnut intake amounts.	⊕⊕⊕O MODERATE	The majority of studies (81%) reporting inflammation outcomes observed no effects of walnut. Additionally, meta-analyses found no significant effects of walnut on inflammation (hsCRP, VCAM, ICAM, TNFa, E-selection, IL-6). Due to concerns of bias and imprecision, the SOE for the effects of walnut intake on inflammation outcomes was rated as *moderate.*
**Glucose homeostasis**								
13	RCTs [40–49,59, 60,62]	Some limitations: Overall ROB was low for 31%, SC for 54%, and high for 15% of the trials reporting glucose outcomes. Studies were limited mostly due to concerns arising from the randomization process, de*via*tions from the intended intervention, missing outcome data, and selection of reported results.	No serious inconsistency: 70% of studies did not find a significant effect of walnut on HbA1c and none of the included studies found an effect of walnut on HOMA-IR.	No serious indirectness: HbA1c and HOMA-IR are validated measures of glucose homeostasis.	Imprecise: Results were imprecise across studies, as indicated by moderate to large statistical heterogeneity in meta-analyses. The 2 studies that could not be meta-analysed also raised concerns of imprecision, due to large measures of variance and lack of quantitative data.	Not applicable; no within study comparisons of different walnut intake amounts.	⊕⊕⊕O MODERATE	70% of RCTs found no significant effect of walnut on HbA1c and no effects were observed on HOMA-IR. The meta-analyses did not find an overall significant effect of walnut on HbA1c or HOMA-IR. Due to concerns of bias and imprecision, SOE for the effects of walnut intake on glucose outcomes was rated as *moderate.*

^a^CI: confidence interval; GRADE: Grades of Recommendation; RCT: randomized-controlled trials; ROB: risk of bias; SC: some concerns; SOE: strength of evidence.

^b^Symbols indicate the following strength of evidence: ⊕⊕⊕⊕, HIGH (further research is very unlikely to change our confidence in the estimate of association); ⊕⊕⊕O, MODERATE (further research is likely to have an important impact on our confidence in the estimate of association and may change the estimate); ⊕⊕OO, LOW (further research is very likely to have an important impact on our confidence in the estimate of association and is likely to change the estimate); and ⊕OOO, VERY LOW (any estimate of association is very uncertain).

## Discussion

The aim of this review was to systematically evaluate existing research on walnuts as a dietary strategy to promote cognitive health and reduce risk for cognitive decline. Previous reviews have described the beneficial effects of walnuts on cognitive function in animal studies, which include improvements in learning, memory, and anxiety behaviours in aged animals [2] and an animal model of Alzheimer’s disease [17] following walnut supplementation. To our knowledge, this is the first systematic review evaluating the effect of walnut intake on cognition-related outcomes in human adults, using evidence from both RCT and observational studies. A strength of this review is that we utilized validated and high methodological standards including PRISMA reporting guidelines, Cochrane ROB 2.0 and NOS to assess risk of bias, the GRADE approach for strength of evidence. While previous reviews have examined the effects of total nut intake [67,68], this review allows isolation of the effects of walnut on cognitive health. Additionally, we included a diversity of study populations and cognition-related outcomes. Our review of cognition-related outcomes included results from five RCT and seven observational study publications, covering broad domains ranging from memory, language, perception, verbal and non-verbal reasoning, as well as mood, structural and functional MRI, and stroke. Owing to the heterogeneity of cognitive tests used, we could not perform a meta-analysis of these outcomes, and findings were instead summarized by outcome and study type.

In the majority of the included RCTs, there was no significant overall effect of walnut intake on cognition-related outcomes (cognitive function and mood), although there were a few exceptions. In the two RCTs reporting cognitive function outcomes, significant effects of 30–60 g/day of walnuts were found on subdomains of cognition and/or subgroups of study populations, although not on total cognitive scores. In the WAHA study [37], participants in the walnut arm had improved global cognition and perception scores after two-years, although only in the Barcelona subgroup. Pribis et al. showed an improvement in a subdomain of verbal reasoning (inference) for participants in the walnut arm as compared to control [35]. Of the three studies reporting mood outcomes, only one study found that 60 g/day of walnuts improved total mood scores compared to control, although only in males [36]. In addition to a limited number of studies reporting cognitive function outcomes, overall risk of bias was rated as some concerns or high for the included studies, mostly due to issues arising from the randomization process, deviations from intended interventions, or outcome reporting.

In contrast to results from RCTs, the majority of the seven included observational studies found significant associations and a dose-response relationship between walnut intake and cognition-related outcomes. Most of these studies were of cross-sectional design but used large pre-existing datasets. In analyses of both the NHS and HPFS cohorts, higher walnut intake was related to lower risk of stroke [20,33], and evaluation of the NHANES cohort data found an inverse association between walnut consumption and depression scores [29]. Additionally, walnut intake was associated with improved performance on a battery of cognitive function tests in both younger and older adults of NHANES cohorts [30] but only with higher working memory scores in older adults enrolled in the PREDIMED study [32]. An analysis of participants from the NHS study [34] similarly showed a cross-sectional association between higher walnut intake and better verbal memory and global cognitive function, although results showed no relationship with trajectory of cognitive decline over time. A secondary analysis of the HRS and HCN study [31] also showed cross-sectional associations between global cognition and walnut intake, but no relation with change in cognitive function during follow-up.

While these observational studies show more promise for a role of dietary walnut in maintenance of cognitive health than results from RCTs, traditionally, observational studies, and in particular, cross-sectional studies, are thought to provide lower strength-of-evidence than do the gold-standard of RCTs [69]. Additionally, overall ROB was low for only one of the included observational studies, and some concerns for the other six studies, mostly due to participant selection and self-reported walnut intake. The weighting of evidence from observational studies and RCTs remains an issue of serious deliberation, particularly for research in disease prevention and nutrition [70]. Factors such as study population, background diet, dose and duration of the intervention, and compliance need to be taken into account when interpreting results. Fundamental complexities in evaluating dietary intake and maintenance of cognitive function in adulthood can contribute to inconsistencies in results between RCTs and observational studies.

In addition to cognitive function and related outcomes, we aimed to evaluate the effect of walnut on risk factors for cognitive decline, including alterations in blood lipids, glucose metabolism, blood pressure, endothelial function, inflammation, and oxidative stress [2–4,10]. Since systematic reviews and meta-analyses examining the effects of walnuts on blood lipids, blood pressure [20,71], oxidative stress [20] and endothelial function [21,72] have been published prior to the end of our search date (April 2020), we report a systematic review and meta-analysis of measures of inflammation and glucose homeostasis. Our meta-analyses found no significant effect of walnut consumption on HbA1c or HOMA-IR across included studies. However, the heterogeneity observed in the HbA1c meta-analysis was large, and may be due to varying baseline health status of the participants in the ten studies. The four studies included for the HOMA-IR outcome also had moderate statistical heterogeneity, but overall did not show an effect of walnut consumption. These findings concur with a recently published systematic review and meta-analysis, which found no significant effects of walnut consumption on HbA1c and HOMA-IR [73]. Likewise, our meta-analyses for the inflammation markers hsCRP, IL-6, TNFɑ, E-Selectin, sICAM-1 and sVCAM-1 did not reach statistical significance.

Previous systematic reviews and meta-analyses did not show an effect of dietary walnut intake on blood pressure [71,74], or oxidative stress [74]. However, the meta-analysis by Guasch-Ferré et al. [74] found that walnut consumption resulted in significantly higher reduction of total cholesterol, LDL cholesterol, triglycerides, ApoA, and ApoB. Two previous meta-analyses also showed an association between walnut intake and endothelial function, although the effect-size was small and there was no dose-response curve [21,72]. Given the link between damage to brain vasculature and cognitive decline, vascular dementia, and Alzheimer’s disease [75–77] there are plausible biological mechanisms by which incorporating walnuts into a regular diet can promote cognitive health. Our review of risk factors for cognitive decline suggests that walnuts may promote cognitive health by targeting cardiovascular mechanisms, specifically dyslipidemia, rather than other metabolic disturbances. However, additional studies are necessary to investigate the mechanistic role of walnuts on cognition.

Overall, the available evidence reviewed in this report is not sufficient to draw a firm conclusion regarding the effect of walnut intake on either cognitive decline or on risk factors for cognitive decline in adults. The limitations identified in this review impel us to suggest some directions for future research.

First, the heterogeneity in cognitive function measures, and minimal reporting of stroke and mood outcomes ruled out meta-analyses for these outcomes. Future RCTs involving cognitive outcomes should specify a minimal set of tests to be included, and these tests should be sufficiently sensitive to detect small changes in cognition-related outcomes. Second, the included studies showed variability in duration and dosage of interventions, background diet and study population characteristics, which hinders comparability of findings. This heterogeneity precludes firm conclusions on the walnut intake regimen required for cognitive benefit, and which populations are likely to experience these beneficial effects. Future studies should specify a set of standard dosages and durations of the intervention and follow-up. Additionally, measures of risk factors for cognitive decline should be incorporated into studies examining the effects of walnut on cognitive function, to elucidate possible biological mechanisms. Third, trials should focus on “at-risk” individuals (such as those with dyslipidemia), since healthy individuals consuming healthy diets are less likely to show cognitive improvement upon dietary intervention. Similarly, trials on mood outcomes should focus on subjects reporting symptoms at screening. Lastly, wherever realistic, biomarkers of nutrient intake should be used since recall of food intake has long been acknowledged to be a limitation in studies involving self-reported dietary assessments. Self-reported food intake, and the resulting risk of recall bias is of particular concern in studies on cognitive function and decline, as even mild cognitive impairment has been shown to reduce the validity of FFQs [78–80]. Biomarkers specific to walnut intake include α-linolenic acid, urolithins, and 5-hydroxyindole-3-acetic acid [81] and their measurement should be considered where appropriate as objective tools for dietary assessment.

In conclusion, the existing evidence, although with low level of confidence, suggests that walnut intake may have a beneficial effect on cognition-related outcomes, including cognitive function, mood, and stroke. Limitations in study design and comparability render the available evidence insufficient to draw a firm conclusion regarding the effects of walnut on cognition in adults. High quality studies and standardized interventions and measurement tools are necessary to determine the role of walnut intake in cognitive health.

## Supplementary Material

Supplemental Material
