# Supplementary material for: Walnut intake, cognitive outcomes and risk factors: a systematic review and meta-analysis
Source: Ann Med. 2021 Jun 16;53(1):972–98. doi: 10.1080/07853890.2021.1925955 (PMC8211141; doi:10.1080/07853890.2021.1925955)
Supplement: Supplemental Material [file IANN_A_1925955_SM3533.zip › Supplemental files/WalnutSR_AnnalsOfMedicineNutritionSectionSupplementalFile1_12Jan2021.docx]

**Newcastle-Ottawa Scale adapted for cross-sectional studies**

**Selection:** (Maximum 5 stars)

1. Representativeness of the sample:

a) Truly representative of the average ***adult (healthy or at risk for cognitive decline)*** in the target population. * (all subjects or random sampling)

b) Somewhat representative of the average ***adult (healthy or at risk for cognitive decline****)* in the target population.* (non-random sampling)

c) Selected group of users. ***E.g., nurses, volunteers***

d) No description of the sampling strategy.

1. Sample size:

a) Justified and satisfactory. *

b) Not justified.

1. Non-respondents:

a) Comparability between respondents and non-respondents characteristics is established, and the response rate is satisfactory. *

b) The response rate is unsatisfactory, or the comparability between respondents and non-respondents is unsatisfactory.

c) No description of the response rate or the characteristics of the responders and the non-responders.

1. Ascertainment of the exposure ***(walnut intake):***

***a) structured interview * ****

***b) written self-report ****

c) no description

**Comparability:** (Maximum 2 stars)

1. The subjects in different outcome groups are comparable, based on the study design or analysis. Confounding factors are controlled.

a) The study controls for the most important factors*:* ***age, education and sex*. ***

b) The study control for any additional factors such as ***baseline cognitive score, socioeconomic status, metabolic or genetic factors.* ***

**Outcome:** (Maximum 3 stars)

1. Assessment of the outcome:

a) Independent blind assessment, ***using validated cognitive test*** **

b) Record linkage. **

c) Self-administered, ***using a validated test***. *

d) No description ***or non-validated test.***

1. Statistical test:

a) The statistical test used to analyze the data is clearly described and appropriate, and the measurement of the association is presented, including confidence intervals and the probability level (p value). *

b) The statistical test is not appropriate, not described or incomplete.

This form was adapted from the Newcastle-Ottawa Quality Assessment Scale for cohort studies to perform quality assessment of cross-sectional studies by Modesti, et al., *Panethnic Differences in Blood Pressure in Europe: A Systematic Review and Meta-Analysis.* PLoS One, 2016. **11**(1): p. e0147601. In our scale, we modified the ascertainment of exposure to reflect assessment of dietary intake. Two stars were given to assessment via structured interviews, and one star was assigned to written self-report. ***Bolded italics*** indicate changes made to the form used by Modesti et al.
