# Supplementary material for: Walnut intake, cognitive outcomes and risk factors: a systematic review and meta-analysis
Source: Ann Med. 2021 Jun 16;53(1):972–98. doi: 10.1080/07853890.2021.1925955 (PMC8211141; doi:10.1080/07853890.2021.1925955)
Supplement: Supplemental Material [file IANN_A_1925955_SM3533.zip › Supplemental files/WalnutSR_AnnalsOfMedicineNutritionSectionSupplementalTables_12Jan2021.docx]

**Table S1.** Literature Search Strategy

| Database: Ovid MEDLINE(R) <1946 to April Week 2 2020>, EBM Reviews - Cochrane Central Register of Controlled Trials <March 2020>, CAB Abstracts <1910 to 2020 Week 15> | |
| --- | --- |
| Search Strategy: | |
| 1 | depression.mp. or exp Depression/ |
| 2 | depressive symptom$.mp. |
| 3 | mood.mp. or exp Affect/ |
| 4 | EMOTIONs.mp. or exp Emotions/ |
| 5 | exp Performance Anxiety/ or exp Anxiety/ or exp Test Anxiety Scale/ or anxiety.mp. or exp Anxiety Disorders/ |
| 6 | or/1-5 |
| 7 | exp juglans/ or juglans.af. or walnut.tw. or walnut$.tw. |
| 8 | ((walnut$ and extract) or (walnut$ and oil)).tw. |
| 9 | 7 or 8 |
| 10 | Cognitive Disorders/ or (cognition or (cognitive and tests) or (cognition and tests)).tw. |
| 11 | exp oxidative stress/ or (oxidative and stress).af. |
| 12 | exp inflammation/ or inflammation.af. |
| 13 | Stroke$.tw. or Stroke/et or Stroke/ep or Stroke/pc or Stroke/mo |
| 14 | cerebral.tw. or Cerebral Infarction/et or Cerebral Infarction/ep or Cerebral Infarction/pc or Cerebral Infarction/mo |
| 15 | exp cerebrovascular disorder/ or cerebrovascular disorders/et or cerebrovascular disorders/ep or cerebrovascular disorders/pc or cerebrovascular disorders/mo or cerebrovascular.tw. |
| 16 | Metabolic Syndrome X/ep or Metabolic Syndrome X/et or Metabolic Syndrome X/pc |
| 17 | ((serum adj1 cholesterol) or (dyslipid$ or hypolipid$ or hyperlipid$) or (LDL or VLDL or triglyceride$ or HDL)).tw. |
| 18 | (Blood pressure or hypertension).af. or exp hypertension/ |
| 19 | exp diabetes mellitus/ or diabetes.af. |
| 20 | exp blood glucose/ or (blood and glucose).af. or blood glucose/ |
| 21 | exp insulin/ or insulin.af. |
| 22 | exp dementia/ or dementia.af. |
| 23 | exp Alzheimer disease/ or alzheimer.af. |
| 24 | exp cognitive dysfunction/ or (cognitive and impair$).af. or (cognitive and dysfunction).af. |
| 25 | or/10-24 |
| 26 | 9 and 25 |
| 27 | Animals/ not human/ |
| 28 | 26 not 27 |
| 29 | remove duplicates from 28 |
| 31 | 6 and 9 |
| 32 | 31 not 27 |
| 33 | remove duplicates from 32 |
| 34 | 30 or 33 |

**Table S2.** ROB assessment for all included RCTs using the Cochrane’s ROB 2.0 Scale^1^

| **Study (Year)** | **Risk due to** |  |  |  |  |  |  |
| --- | --- | --- | --- | --- | --- | --- | --- |
|  | **1. Randomization process** | **2a. Deviations from intended intervention *(effect of assignment)*** | **2b. Deviations from intended intervention**  ***(effect of adhering)*** | **3. Missing outcome data** | **4. Outcome measurement** | **5. Selection of reported result** | **Overall ROB** |
| **Cognition-related Outcomes** |  |  |  |  |  |  |  |
| Miller et al., 2018 | High | SC | SC | Low | Low | Low | High |
| Pribis et al., 2016 | Low | Low | Low | SC | Low | Low | SC |
| Pribis et al., 2012 | Low | Low | Low | SC | Low | Low | SC |
| Probst et al., 2012 | SC | Low | Low | Low | SC | High | High |
| Sala-Vila et al., 2020 | Low | SC | High | Low | Low | Low | High |
| **Risk-factor Outcomes** |  |  |  |  |  |  |  |
| Aronis et al., 2012 | Low | Low | Low | Low | Low | Low | Low |
| Bamberger et al., 2017 | Low | High | High | Low | Low | SC | High |
| Bhardwaj et al., 2018 | Low | Low | Low | Low | Low | Low | Low |
| Burns-Whitmore et al., 2014 | Low | SC | SC | High | Low | Low | High |
| Canales et al., 2011 | Low | SC | SC | SC | Low | Low | SC |
| Chiang et al., 2012 | SC | SC | High | High | Low | Low | High |
| Cortes et al., 2006 | Low | Low | Low | Low | Low | Low | Low |
| Damasceno et al., 2011 | Low | SC | SC | SC | Low | Low | SC |
| Fatahi et al., 2019 | Low | Low | Low | Low | Low | Low | Low |
| Holscher et al., 2018 | Low | Low | Low | Low | Low | SC | Low |
| Hwang et al., 2019 | SC | SC | Low | SC | Low | Low | SC |
| Katz et al., 2012 | Low | Low | SC | High | Low | Low | High |
| Ma et al., 2010 | SC | SC | SC | SC | Low | Low | SC |
| Mukuddem-Petersen et al., 2007 | Low | Low | Low | Low | Low | Low | Low |
| Njike et al., 2015 | Low | Low | Low | High | Low | SC | SC |
| Pieters et al., 2005 | Low | Low | Low | Low | Low | Low | Low |
| Rock et al., 2016 | Low | SC | Low | High | Low | Low | SC |
| Ros et al., 2004 | Low | Low | Low | Low | Low | Low | Low |
| Tapsell et al., 2009 | Low | SC | Low | Low | Low | Low | SC |
| Tapsell et al., 2004 | SC | Low | Low | Low | Low | Low | Low |
| Tapsell et al., 2017 | Low | Low | Low | Low | Low | Low | Low |
| Tindall et al., 2019 | SC | SC | Low | High | Low | Low | SC |
| Wu et al., 2014 | SC | SC | High | High | Low | SC | SC |
| Wu et al., 2010 | Low | Low | Low | Low | Low | Low | Low |
| Zhao et al., 2004 | SC | SC | Low | Low | Low | Low | SC |
| Zhao et al., 2007 | SC | Low | Low | Low | Low | Low | SC |
| Zibaeenezhad et al., 2016 | Low | SC | SC | SC | Low | Low | SC |

^1^RCT, randomized-controlled trial; ROB, risk of bias; SC, some concerns

**Table S3.** List of 53 Publications Identified from Abstract Screening but Excluded After Full-text Screening

| PMID | Title | Author | Journal | Year | Reason for Exclusion |
| --- | --- | --- | --- | --- | --- |
| None | Short-term walnut consumption does not affect markers of inflammation and vascular injury in obese humans with the metabolic syndrome | Aronis KN, Magkos F, Vamvini MT, Sweeney LL, Brennan A, Mantzoros CS. | The Endocrine Society's 93rd Annual Meeting | 1993 | Duplicate- Abstract of included data |
| 23422923 | The relationship between nut consumption and lipid profile among the Iranian adult population; Isfahan Healthy Heart Program | Askari G, Yazdekhasti N, Mohammadifard N, Sarrafzadegan N, Bahonar A, Badiei M, Sajjadi F, Taheri M. | Eur J Clin Nutr. | 2013 | Walnut intake not calculable |
| 25690866 | Weight loss with a modified Mediterranean-type diet using fat modification: a randomized controlled trial | Austel A, Ranke C, Wagner N, Gorge J, Ellrott T. | Eur J Clin Nutr. | 2015 | Walnut intake not calculable |
| 28984822 | A Walnut-Enriched Diet Reduces Lipids in Healthy Caucasian Subjects, Independent of Recommended Macronutrient Replacement and Time Point of Consumption: a Prospective, Randomized, Controlled Trial. | Bamberger C, Rossmeier A, Lechner K, Wu L, Waldmann E, Stark RG, Altenhofer J, Henze K, Parhofer KG. | Nutrients. | 2017 | Duplicate |
| None | Walnut-enriched diet reduces lipids in healthy caucasian subjects, independent of recommended macronutrient replacement | Bamberger C, Rossmeier A, Lechner K, Wu L, Waldmann E, Stark RG, Altenhofer J, Henze K, Parhofer K. | J Am Coll Cardiol. | 2017 | Duplicate- Abstract of included data |
| 27568885 | Adherence to a Mediterranean-Style Diet and Its Influence on Cardiovascular Risk Factors in Postmenopausal Women | Bihuniak JD, Ramos A, Huedo-Medina T, Hutchins-Wiese H, Kerstetter JE, Kenny AM. | J Acad Nutr Diet. | 2016 | Co-intervention |
| 24673793 | Effects of supplementing n-3 fatty acid enriched eggs and walnuts on cardiovascular disease risk markers in healthy free-living lacto-ovo-vegetarians: a randomized, crossover, free-living intervention study. | Burns-Whitmore B, Haddad E, Sabate J, Rajaram S. | Nutr J. | 2014 | Duplicate |
| 22959058 | Postprandial changes in the proteome are modulated by dietary fat in patients with metabolic syndrome | Camargo A, Rangel-Zuniga OA, Pena-Orihuela P, Marin C, Perez-Martinez P, Delgado-Lista J, Gutierrez-Mariscal FM, Malagon MM, Roche HM, Tinahones FJ, Perez-Jimenez F, Lopez-Miranda J. | J Nutr Biochem. | 2013 | Walnut intake not calculable |
| 28715141 | Walnut consumption increases activation of the insula to highly desirable food cues: A randomized, double-blind, placebo-controlled, cross-over fMRI study | Farr OM, Tuccinardi D, Upadhyay J, Oussaada SM, Mantzoros C. | Diabetes Obes Metab. | 2018 | Duration too short |
| 17125536 | Tree nuts and the lipid profile: a review of clinical studies. | Griel AE, Kris-Etherton P. | Br J Nutr. | 2006 | Review paper |
| 18952211 | Olive oil and walnut breakfasts reduce the postprandial inflammatory response in mononuclear cells compared with a butter breakfast in healthy men | Jimenez-Gomez Y, Lopez-Miranda J, Blanco-Colio LM, Marin C, Perez-Martinez P,Ruano J,Paniagua JA, Rodriguez F, Egido J, Perez-Jimenez J. | Atherosclerosis. | 2009 | Co-intervention |
| 10090861 | Blood cholesterol and walnut consumption: a cross-sectional survey in France | Lavedrine F, Zmirou D, Ravel A, Balducci F,Alary J. | Prev Med. | 1999 | Walnut intake not calculable |
| 26811166 | Effects of Diet Composition and Insulin Resistance Status on Plasma Lipid Levels in a Weight Loss Intervention in Women. | Le T, Flatt SW, Natarajan L, Pakiz B, Quintana EL, Heath DD, Rana BK, Rock CL. | J Am Heart Assoc. | 2016 | Duplicate |
| None | The healthtrack study- A randomised control trial of interdisciplinary care on weight loss 12 month follow up | Lonergan MA, Murali K, Tapsell LC,Batterham MJ, Neale EP, Martin A,Thorne R, Deane F, Peoples G. | ANZSN ASM 2018. | 2017 | Duplicate - Abstract of included data |
| None | Effect of apples and walnuts on classical risk factors and inflammatory markers in patients after myocardial infarction undergoing cardiac rehabilitation | Mlakar P, Salobir B, Strasek J, Eobo N, Tereelj M, Sabovic M. | Ann Nutr Metab. | 2013 | Co-intervention |
| 26688734 | Walnut ingestion in adults at risk for diabetes: effects on body composition, diet quality, and cardiac risk measures. | Njike VY, Ayettey R, Petraro P, Treu JA, Katz DL. | BMJ Open Diabetes Res Care. | 2015 | Duplicate |
| 26813890 | Dietary alpha-Linolenic Acid, Marine omega-3 Fatty Acids, and Mortality in a Population With High Fish Consumption: Findings From the PREvencion con DIeta MEDiterranea (PREDIMED) Study.[Erratum appears in J Am Heart Assoc. 2016 Feb;5(2). pii: e002077. doi: 10.1161/JAHA.116.002077; PMID: 26873691] | Sala-Vila A, Guasch-Ferre M, Hu FB, Sanchez-Tainta A, Bullo M, Serra-Mir M, Lopez-Sabater C, Sorli JV, Aros F, Fiol M, Munoz MA, Serra-Majem L, Martinez JA, Corella D, Fito M, Salas-Salvado J, Martinez-Gonzalez MA, Estruch R, Ros E; PREDIMED I,B. | J Am Heart Assoc. | 2016 | Co-intervention |
| 12433513 | Effect of an Indo-Mediterranean diet on progression of coronary artery disease in high risk patients (Indo-Mediterranean Diet Heart Study): a randomised single-blind trial | Singh RB, Dubnov G, Niaz MA, Ghosh S, Singh R, Rastogi SS, Manor O, Pella D, Berry EM. | Lancet | 2002 | Co-intervention |
| 28710205 | Effect of interdisciplinary care on weight loss: a randomised controlled trial. | Tapsell LC, Lonergan M, Batterham MJ, Neale EP, Martin A, Thorne R, Deane F, Peoples G. | BMJ Open. | 2017 | Duplicate |
| 28814394 | Dynamics of intrapericardial and extrapericardial fat tissues during long-term, dietary-induced, moderate weight loss | Tsaban G, Wolak A, Avni-Hassid H, Gepner Y, Shelef I, Henkin Y, Schwarzfuchs D, Cohen N, Bril N, Rein M, Serfaty D, Kenigsbuch S, Tene L, Zelicha H, Yaskolka-Meir A, Komy O, Bilitzky A, Chassidim Y, Ceglarek U, Stumvoll M, Bluher M, Thiery J, Dicker D, Rudich A, Stampfer MJ, Shai I. | Am J Clin Nutr. | 2017 | Co-intervention |
| None | Walnut-enriched diet reduces fasting non-HDL-cholesterol in healthy caucasian subjects | Wu L, Piotrowski K, Rau T, Waldmann E, Broedl UC, Mantzoros C, Parhofer KG. | Circulation. | 2018 | Duplicate- Abstract of included data |
| 28394361 | Effects of walnut oil on lipid profiles in hyperlipidemic type 2 diabetic patients: a randomized, double-blind, placebo-controlled trial. | Zibaeenezhad MJ, Farhadi P, Attar A, Mosleh A, Amirmoezi F, Azimi A. | Nutr Diabetes. | 2017 | Duplicate |
| 29552423 | Insulin Resistance Improves More in Women than In Men in Association with a Weight Loss Intervention | Badri NW, Flatt SW, Barkai HS, Pakiz B, Heath DD , Rock CL. | J Obes Weight Loss Ther. | 2018 | Co-intervention |
| 31003626 | Effect of a high-fat Mediterranean diet on bodyweight and waist circumference: a prespecified secondary outcomes analysis of the PREDIMED randomised controlled trial | Estruch R, Martinez-Gonzalez MA, Corella D, Salas-Salvado J, Fito M, Chiva-Blanch G, Fiol M, Gomez-Gracia E, Aros F, Lapetra J, Serra-Majem L, Pinto X, Buil-Cosiales P, Sorli JV, Munoz M, Basora-Gallisa J, Lamuela-Raventos RM, Serra-Mir M, Ros E. | Lancet Diabetes Endocrinol. | 2019 | Co-intervention |
| 30518050 | Walnut Consumption for Two Years and Leukocyte Telomere Attrition in Mediterranean Elders: results of a Randomized Controlled Trial | Freitas-Simoes TM, Cofan M, Blasco M, Soberon N, Foronda M, Serra-Mir , Roth I, Valls-Pedret C, Domenech M, Ponferrada-Ariza E, Calvo C, Rajaram S, Sabate J, Ros E, Sala-Vila A. | Nutrients. | 2018 | No outcomes of interest |
| 31075323 | The beneficial effects of Mediterranean diet over low-fat diet may be mediated by decreasing hepatic fat content | Gepner Y, Shelef I, Komy O, Cohen N, Schwarzfuchs , Bril N, Rein M, Serfaty D, Kenigsbuch S, Zelicha H, Yaskolka Meir, Tene L, Bilitzky A, Tsaban G, Chassidim Y, Sarusy B, Ceglarek U, Thiery J, Stumvoll M, Bluher M, Stampfer MJ, Rudich A, Shai I. | J Hepatol. | 2019 | Co-intervention |
| 29142011 | Effect of Distinct Lifestyle Interventions on Mobilization of Fat Storage Pools: CENTRAL Magnetic Resonance Imaging Randomized Controlled Trial | Gepner Y, Shelef I, Schwarzfuchs D, Zelicha H, Tene L, Yaskolka Meir A, Tsaban G, Cohen N, Bril N, Rein M, Serfaty D, Kenigsbuch S, Komy O, Wolak A, Chassidim Y, Golan R, Avni-Hassid H, Bilitzky A, Sarusi B, Goshen E, Shemesh E, Henkin Y, Stumvoll M, Bluher M, Thiery , Ceglarek U, Rudich A, Stampfer MJ, Shai I. | Circulation. | 2018 | Co-intervention |
| 29931130 | Effects of walnut consumption on blood lipids and other cardiovascular risk factors: an updated meta-analysis and systematic review of controlled trials | Guasch-Ferre M, Li J, Hu FB, Salas-Salvado J, Tobias DK. | Am J Clin Nutr. | 2018 | Meta-analysis/ Systematic review |
| 29145952 | Nut Consumption and Risk of Cardiovascular Disease | Guasch-Ferre M, Liu X, Malik VS, Sun Q, Willett WC, Manson JE, Rexrode KM, Li Y, Hu FB, Bhupathiraju SN. | J Am Coll Cardiol. | 2017 | Duplicate |
| 28946607 | Prospective Study of Nut Consumption and Incidence of Metabolic Syndrome: Tehran Lipid and Glucose Study | Hosseinpour-Niazi S, Hosseini S, Mirmiran P, Azizi F. | Nutrients. | 2017 | Duplicate |
| 30271610 | Pilot randomized controlled trial of a Mediterranean diet or diet supplemented with fish oil, walnuts, and grape juice in overweight or obese US adults | Jaacks LM, Sher S, Staercke , Porkert M, Alexer WR, Jones D, Vaccarino V, Ziegler TR, Quyyumi AA. | BMC Nutr. | 2018 | Co-intervention |
| 31773150 | Comparative effects of different types of tree nut consumption on blood lipids: a network meta-analysis of clinical trials | Liu K, Hui S, Wang B, Kaliannan K, Guo X, Liang L. | Am J Clin Nutr. | 2020 | Meta-analysis |
| 29865169 | Mastication of nuts under realistic eating conditions: implications for energy balance | McArthur BM, Considine RV, Mattes RD. | Nutrients. | 2018 | Duration too short |
| 30068215 | The Resulting Variation in Nutrient Intake With the Inclusion of Walnuts in the Diets of Adults at Risk for Type 2 Diabetes: a Randomized, Controlled, Crossover Trial | Njike V, Costales V, Petraro P, Annam R, Yar,i , Katz DL. | Am J Health Promot. | 2019 | No outcomes of interest |
| 31196252 | Effects of nut and seed consumption on markers of glucose metabolism in adults with prediabetes: a systematic review of randomised controlled trials | Ntzouvani A, Antonopoulou S, Nomikos T. | Br J Nutr. | 2019 | Systematic review |
| 28119602 | The Walnuts and Healthy Aging Study (WAHA): protocol for a nutritional intervention trial with walnuts on brain aging | Rajaram S, Valls-Pedret C, Cofan M, Sabate J, Serra-Mir M, Perez-Heras A, Arechiga, Casaroli-Marano RP, Alforja , Sala-Vila , Domenech , Roth , Freitas-Simoes TM, Calvo, Lopez-Illamola A, Haddad, Bitok E, Kazzi N, Huey L, Fan J, Ros E. | Front Aging Neurosci. | 2017 | Methods paper |
| 29064409 | Effect of Altering Dietary n-6:n-3 Polyunsaturated Fatty Acid Ratio with Plant and Marine-Based Supplement on Biomarkers of Bone Turnover in Healthy Adults | Rajaram S, Yip EL, Reghunathan R, Mohan S, Sabate J. | Nutrients. | 2017 | Co-intervention |
| 28555011 | The IL-6 Gene Promoter SNP and Plasma IL-6 in Response to Diet Intervention | Rana BK, Flatt SW, Health DD, Pakiz B, Quintana EL, Natarajan L, Rock CL. | Nutrients. | 2017 | Co-intervention |
| 28610906 | A walnut-containing meal had similar effects on early satiety, CCK, and PYY, but attenuated the postprandial GLP-1 and insulin response compared to a nut-free control meal | Rock CL, Flatt SW, Barkai HS, Pakiz B, Heath DD. | Appetite. | 2017 | Duration too short |
| 29202751 | Walnut consumption in a weight reduction intervention: effects on body weight, biological measures, blood pressure and satiety | Rock CL, Flatt SW, Barkai HS, Pakiz B, Heath DD. | Nutr J. | 2017 | Duplicate |
| 31881702 | A Pilot Randomized Crossover Trial Assessing the Safety and Short-Term Effects of Walnut Consumption by Patients with Chronic Kidney Disease | Sanchis P, Molina M, Berga F, Munoz E, Fortuny R, Costa-Bauza A, Grases F, Buades JM. | Nutrients. | 2019 | Patients with CKD |
| 28347564 | The gut microbiota metabolism of pomegranate or walnut ellagitannins yields two urolithin-metabotypes that correlate with cardiometabolic risk biomarkers: Comparison between normoweight, overweight-obesity and metabolic syndrome | Selma MV, Gonzalez-Sarrias A, Salas-Salvado J, Andres-Lacueva C, Alasalvar C, Orem A, Tomas-Barberan F A, Espin JC. | Clin Ntr. | 2018 | No outcomes of interest |
| 29576369 | The effect of long-term weight-loss intervention strategies on the dynamics of pancreatic-fat and morphology: an MRI RCT study | Tene L, Shelef I, Schwarzfuchs D, Gepner Y, Yaskolka Meir A, Tsaban G, Zelicha H, Bilitzky A, Komy O, Cohen N, Bril N, Rein M, Serfaty D, Kenigsbuch S, Chassidim Y, Sarusy B, Ceglarek U, Stumvoll M , Bluher M, Thiery J, Stampfer MJ, Rudich A, Shai I. | Clin Nutr ESPEN. | 2018 | Co-intervention |
| 31848609 | Walnuts and Vegetable Oils Containing Oleic Acid Differentially Affect the Gut Microbiota and Associations with Cardiovascular Risk Factors: Follow-up of a Randomized, Controlled, Feeding Trial in Adults at Risk for Cardiovascular Disease | Tindall AM, McLimans CJ, Petersen KS, Kris-Etherton PM, Lamendella R. | J Nutr. | 2019 | No outcomes of interest |
| None | Walnuts and Vegetable Oils Differentially Affect the Gut Microbiome and Associations with Cardiovascular Risk Factors (OR29-06-19) | Tindall A, McLimans C, Petersen K, Kris-Etherton P, Lamendella R. | Current Developments in Nutrition | 2019 | No outcomes of interest |
| 4584788 | Depression of lymphocyte transformation and exacerbation of Behcet's syndrome by ingestion of english walnuts. | Marquardt JL, Snyderman R, Oppenheim JJ. | Cellular Immunology. | 1973 | No outcomes of interest |
| 32053984 | Effects of supplementing the usual diet with a daily dose of walnuts for two years on metabolic syndrome and its components in an elderly cohort | Abdrabalnabi AA, Rajaram S, Bitok E, Oda K, Beeson WL, Kaur A, Cofan M, Serra-Mir M, Roth I, Ros E, Sabate J. | Nutrients. | 2020 | No outcomes of interest |
| 31909809 | Replacing Saturated Fats with Unsaturated Fats from Walnuts or Vegetable Oils Lowers Atherogenic Lipoprotein Classes Without Increasing Lipoprotein(a) | Tindall AM, Kris-Etherton PM, Petersen KS. | Journal of nutrition. | 2020 | No outcomes of interest |
| None | Effects of 2-year walnut supplementation on cognitive decline in healthy elders: the walnuts and healthy aging (WAHA) study | Coll-Padros N, Sala-Vila A, Valls-Pedret C, Serra-Mir M, Cofan M, Roth I, Freitas-Simoes T, Domenech M, Vaque-Alcazar L, Bartres-Faz D, Rajaram S, Sabate J, Ros E. | Journal of prevention of alzheimer's disease. | 2018 | Duplicate |
| None | The healthtrack study- A randomised control trial of interdisciplinary care on weight loss 12 month follow up | Lonergan MA, Murali K, Tapsell LC, Batterham MJ, Neale EP, Martin A, Thorne R, Deane F, Peoples G. | Nephrology | 2017 | Abstract |
| 27792133 | Effects of Walnut Consumption on Mood in Young Adults-A Randomized Controlled Trial | Pribis P. | Nutrients. | 2016 | Duplicate |
| None | Effects of walnut consumption on mood in young adults. | Pribis P, Bailey R, Hernandez M, Grajales T, Sabate J. | FASEB journal. (Meeting Abstracts) | 2015 | Abstract of included study |
| 16733237 | Modulation of baroreflex sensitivity by walnuts versus cashew nuts in subjects with metabolic syndrome | Schutte AE, Van Rooyen JM, Huisman HW, Mukuddem-Petersen J, Oosthuizen W, Hanekom SM, Jerling JC | Am J Hypertens | 2006 | Duplicate of included study |
